# A novel necroptosis-related gene index for predicting prognosis and a cold tumor immune microenvironment in stomach adenocarcinoma

**DOI:** 10.3389/fimmu.2022.968165

**Published:** 2022-10-27

**Authors:** Muhammad Khan, Jie Lin, Baiyao Wang, Chengcong Chen, Zhong Huang, Yunhong Tian, Yawei Yuan, Junguo Bu

**Affiliations:** ^1^ Department of Oncology, Guangdong Second Provincial General Hospital, Guangzhou, China; ^2^ Department of Radiation Oncology, Affiliated Cancer Hospital and Institute of Guangzhou Medical University, Guangzhou, China

**Keywords:** programmed cell death, tumor microenvironment, molecular targeted therapy, immunotherapy, cancer prognosis

## Abstract

**Background:**

Gastric cancer (GC) represents a major global clinical problem with very limited therapeutic options and poor prognosis. Necroptosis, a recently discovered inflammatory form of cell death, has been implicated in carcinogenesis and inducing necroptosis has also been considered as a therapeutic strategy.

**Objective:**

We aim to evaluate the role of this pathway in gastric cancer development, prognosis and immune aspects of its tumor microenvironment.

**Methods and results:**

In this study, we evaluated the gene expression of 55 necroptosis-related genes (NRGs) that were identified *via* carrying out a comprehensive review of the medical literature. Necroptosis pathway was deregulated in gastric cancer samples (n=375) as compared to adjacent normal tissues (n=32) obtained from the “The Cancer Genome Atlas (TCGA)”. Based on the expression of these NRGs, two molecular subtypes were obtained through consensus clustering that also showed significant prognostic difference. Differentially expressed genes between these two clusters were retrieved and subjected to prognostic evaluation *via* univariate cox regression analysis and LASSO cox regression analysis. A 13-gene risk signature, termed as necroptosis-related genes prognostic index (NRGPI), was constructed that comprehensively differentiated the gastric cancer patients into high- and low-risk subgroups. The prognostic significance of NRGPI was validated in the GEO cohort (GSE84437: n=408). The NRGPI-high subgroup was characterized by upregulation of 10 genes (CYTL1, PLCL1, CGB5, CNTN1, GRP, APOD, CST6, GPX3, FCN1, SERPINE1) and downregulation of 3 genes (EFNA3, E2F2, SOX14). Further dissection of these two risk groups by differential gene expression analysis indicated involvement of signaling pathways associated with cancer cell progression and immune suppression such as WNT and TGF-β signaling pathway. Para-inflammation and type-II interferon pathways were activated in NRGPI-high patients with an increased infiltration of Tregs and M2 macrophage indicating an exhausted immune phenotype of the tumor microenvironment. These molecular characteristics were mainly driven by the eight NRGPI oncogenes (CYTL1, PLCL1, CNTN1, GRP, APOD, GPX3, FCN1, SERPINE1) as validated in the gastric cancer cell lines and clinical samples. NRGPI-high patients showed sensitivity to a number of targeted agents, in particular, the tyrosine kinase inhibitors.

**Conclusions:**

Necroptosis appears to play a critical role in the development of gastric cancer, prognosis and shaping of its tumor immune microenvironment. NRGPI can be used as a promising prognostic biomarker to identify gastric cancer patients with a cold tumor immune microenvironment and poor prognosis who may response to selected molecular targeted therapy.

## Introduction

Gastric cancer represents a major clinical problem and is ranked fifth for incidence and fourth for mortality globally. It is responsible for 1,089,103 new cases in 2020 and an estimated 768,793 deaths which means one in every 13 deaths (1). The overall survival remains at 31% within the United States and 25% worldwide (2). Pathogenic infections such as H. pylori, which is infecting 50% of global population, and Epstein Barr virus (EBV) have been linked to gastric cancer (3, 4). Eradication of H. pylori strategies have helped to prevent a significant proportion of gastric cancer (5). Multidisciplinary approach of surgery and chemotherapy has improved the survival rate to 60% and 80% in early staged gastric cancer. However, majority of the cases are diagnosed at an advanced stage for whom the 5-year survival rate is merely 18% to 50% (3). These figures indicate the need for more effective molecularly driven treatment strategies.

Necroptosis is a programmed lytic cell death pathway and is deregulated in various inflammatory disorders and cancers (6–8). Receptor-interacting serine/threonine-protein kinase 1 (RIPK1), receptor-interacting protein kinase 3 (RIPK3), and mixed lineage kinase domain like pseudokinase (MLKL), which constitutes the core components of necroptosis, are downregulated in various types of cancers including colorectal cancer, pancreatic adenocarcinoma, cervical squamous cell carcinoma, and melanoma (6, 7, 9–13). Downregulation correlated with histological grade and was shown to be an independent prognostic factor for overall survival (OS) and disease-free survival (DFS) (10). Moreover, testing of more than 60 cancer cell lines showed absence of RIPK3 protein expression in two-thirds of these cancer cell lines, which was restored upon treatment with the hypomethylating agent decitabine (14). In glioblastoma cells, RIPK3 downregulation or inhibition of RIPK1 with Nec-1 were sufficient to abrogate the necroptosis-mediated cell death induced by edelfosine (15). Induction of osteosarcoma cell death *via* necroptosis has also been demonstrated by combining the stress-inducing agents and nuclear factor-kappa B (NF-κB) inhibitors (16). These studies indicate that necroptosis-induction strategies could be exploited for cancer therapy. Necroptosis is characterized by simultaneous swelling of organelles and disruption of plasma membrane leading to organelle breakdown and leakage of intracellular contents resulting in a pro-inflammatory response (7, 8). Carcinogenic effects include its ability to induce inflammation which has been associated with cancer metastasis and T cell death (11). On the other hand, due to its inflammatory nature, necroptosis is also regarded as a potential target for cancer therapy as necroptosis cells were shown to initiate adaptive immunity by activating CD8+ T cells *via* production of antigens and inflammatory stimuli for dendritic cells (DCs) (17). Therefore, understanding the molecular dynamics of this pathway in gastric cancer may unravel its potential as a therapeutic target.

In this study we investigated the role of necroptosis in stomach adenocarcinoma by first 1) reviewing the medical literature for the various molecules involved in the process of necroptosis and then 2) carrying out comprehensive bioinformatic analysis of the expression datasets. We successfully developed a risk model that can not only predict the prognosis of gastric cancer patients but also their immune landscape.

## Materials and methods

### Datasets

Clinical and mRNA expression data of 375 stomach adenocarcinoma tissues (STAD) and 32 adjacent normal tissues was retrieved from TCGA Data Portal (https://portal.gdc.cancer.gov/repository/). Likewise, RNA-Seq and clinical data for external validation cohort (GEO ID: GSE84437) were downloaded from GEO database (https://www.ncbi.nlm.nih.gov/geo/). Both datasets were subjected to log2(x+1) transformation and normalization with “limma” package. Batch effects were removed with “sva” package using “Combat” function. General characteristics of the two cohorts are outlined in **Supplementary Table 1** (**Supplementary Data-Sheet#1**). Genetic alterations data (Simple Nucleotide Variation) of TCGA STAD cohort was downloaded from the University of California, Santa Cruz (UCSC) Xena website (https://xenabrowser.net/). The oncoplot was constructed using the R package “maftools” to analyze the number and categories of gene mutations in two NRGPI subgroups. Protein-protein interaction (PPI) network was constructed with the Search Tool for the Retrieval of Interacting Genes (STRING) database, version 11.5 (https://string-db.org/). STRING is a database of known and predicted protein-protein interactions. Interactions in STRING are derived from five main sources: genomic context predictions; high-throughput lab experiments; (conserved) co-expression; automated textmining; and previous knowledge in databases. The TIMER 2.0 website (http://timer.comp-genomics.org/) was utilized for estimation of association between NRGPI risk genes and macrophage infiltration.

### Identification of necroptosis-related genes

A total of 55 necroptosis-related genes (NRGs) were extracted from The Molecular Signatures Database (*MSigDB*) and previously published reviews and research articles (**Supplementary Data-Sheet#2: Supplementary Table 2**) (18–58). RIPK1, RIPK3 and MLKL constitutes the core components of this pathway. Mainly three receptor pathways were identified in regulation of necroptosis, namely: tumor necrosis factor receptor 1 (TNFR1), FAS receptor (TNFR6) and toll-like receptors (TLR3/4) (30–33). Moreover, negative and positive regulators of RIPK1, RIPK3, MLKL and necrosome (RIPK1-RIPK3-MLKL complex) were also studied (18–58). Differentially expressed genes (DEGs) were determined with the application of “limma” package with p value <0.05.

### Consensus clustering

Consensus clustering analysis, which is a rigorous unsupervised classification technique, was carried out to identify distinct necroptosis patterns based on the expression of 55 NRGs. R package “ConsensuClusterPlus” was utilized for measuring similarity between and within each group *via* Euclidean distance with 1000 times repetition (59). Optimum cluster number (k) and level of consensus stability was determined according to the cumulative distribution function (CDF) plots and the atness of the CDF curve, respectively. Overall survival difference between the clusters was obtained using the R package “survival”. Survival risk was estimated using Cox Proportional-Hazards model and statistical difference was assessed by log-rank test. The DEGs between the clusters were estimated with “limma” package according to the criteria: log fold change (logFC) = 1, and the false discover rate (FDR) < 0.01.

### Development and validation of necroptosis-related prognostic model

Next, univariate cox regression analysis was carried out to estimate the prognostic significance of the DEGs (n=1056) identified between the clusters. In total, 124 DEGs showed prognostic association when significance level was set at p<0.01. Using the R package “glmnet”, these DEGs were subjected to the least absolute shrinkage and selection operator (LASSO) penalized Cox regression analysis to construct the prognostic model by narrowing down the candidate genes (60). Normalized candidate DEGs expression and survival data (time and status) constituted the independent and independent variable of the LASSO regression, respectively. Penalty parameter (λ) was determined with the minimum criteria by using a ten-fold cross-validation. The risk score was calculated for each patient according to the expression level of DEGs and their corresponding coefficient. The formula was as follows: risk score = (expression of mRNA_1_ × coefficient_mRNA1_) + (expression of mRNA_2_ × coefficient_mRNA2_) + … + (expression of mRNA_n_ × coefficient_mRNAn_). The median risk score was used to determine the subgroups into low- and high-risk cohorts. The Kaplan-Meier analysis was performed to compare the overall survival between the risk groups. Receiver operating characteristic (ROC) curve to evaluate diagnostic efficacy of the risk model was obtained *via* ROC curve analysis using the R packages “survival”, “survminer” and “time-ROC”. Furthermore, principal component analysis (PCA) and t-distributed stochastic neighbor embedding (t-SNE) were also performed to further visualize spatial dimensions between the risk groups. PCA was performed using the “prcomp” function in the “stats” R package and t-SNE with the R package “Rtsne”. All of the steps were repeated for validation in the GEO cohort.

### Independent prognostic analysis

We further sought to validate the prognostic model by undertaking independent prognostic analysis in the TCGA and GEO cohorts along with other variables such age, gender, tumor grade and tumor stage (TNM staging data). Univariate and multivariate cox regression models were employed.

### Functional enrichment analysis

Differential expression of genes was investigated between the low- and high-risk subgroups in the TCGA cohort to assess the biological processes and pathways differentiating the risk subgroups. DEGs were filtered according to logFC = 1 and FDR < 0.05. Gene ontology (GO) and Kyoto Encyclopedia of Genes and Genomes (KEGG) analysis was performed based on these DEGs by applying the “clusterProfiler” package. Immune landscape was evaluated in term of immune cell infiltration as well as the immune-related pathways. Infiltration of major immune cells and the status of immune-related pathways was evaluated by employing the “gsva” package to conduct the single-sample gene set enrichment analysis (ssGSEA). Score for the activity of each immune-related pathways was calculated. Furthermore, to elucidate on the subtypes of various immune cells, a quantitative analysis of the relative abundance of 22 types of immune cells in the TCGA cohort was achieved using the CIBERSORT algorithm. The results were used to quantify the difference in the infiltration of each cell in the risk groups. Moreover, the prognostic significance of each immune cell and immune-related pathway was also elaborated by employing the K-M survival analysis. Significance level was set at p<0.05. In an attempt to establish the immune subtype of the risk groups, we used the immune subtype information available from the previous paper to establish the enrichment of each subtype in the high- and low-risk groups (61).

### Construction of competing endogenous RNA network

To construct the competing endogenous RNA network, the miRNA targets for the risk genes were predicted using miRWalk 3.0 (http://mirwalk.umm.uni-heidelberg.de/), which provides the predicted and experimentally verified results of TargetScan, MirTarbase and miRDB. A cutoff criterion (≥ 0.95) was set for the prediction analysis in miRWalk. The miRNA targets obtained were further screened for negative correlation with the risk genes in the TCGA STAD cohort which was followed by further prognostic significance. The starbase v2.0 (http://starbase.sysu.edu.cn/) was investigated for miRNA-lncRNA targets which were then screened for their positive correlation with risk genes and negative correlation with miRNA targets in the TCGA STAD cohort (62). The ceRNA network was plotted with Cytoscape v3.6.0 (63). Correlation scrutiny was tested with Spearman’s correlation test with the following criteria: R=0.2 and p value <0.001.

### Immunohistochemistry

Formalin-fixed, paraffin-embedded 4-μm thick tumor tissue sections were deparaffinized in xylene and ethanol. Antigen retrieval was performed by boiling in a microwave oven (citrate buffer, pH 6.0) which was followed by blocking of endogenous HRP activity with 0.3% hydrogen peroxide. After washing with 10% phosphate buffered saline (PBS), the sections were blocked with 5% BSA and incubated with primary antibodies against RIPK1 (Proteintech, #17519-1-AP, Rabbit, 1:50), RIPK3 (Proteintech, #17563-1-AP, Rabbit, 1:100), MLKL (Proteintech, #21066-1-AP, Rabbit, 1:50), SERPINE1 (Proteintech, #13801-1-AP, Rabbit, 1:50), FCN1 (Proteintech, #11775-1-AP, Rabbit, 1:50), CNTN1 (Proteintech, #13843-1-AP, Rabbit, 1:50), CYTL1 (Proteintech, #15856-1-AP, Rabbit, 1:50), PLCL1 (Abcam, #EPR11213, Rabbit, 1:100), GRP (Proteintech, #28482-1-AP, Rabbit, 1:500), GPX3 (Affinity Biosciences, #DF6765, Rabbit, 1:50), APOD (Proteintech, #10520-1-AP, Rabbit, 1:50), TGFB1 (Affinity Biosciences, #AF1027, Rabbit, 1:100), TGFB3 (Proteintech, #18942-1-AP, Rabbit, 1:50), WNT2B (Affinity Biosciences, #DF12538, Rabbit, 1:100), WNT9A (Affinity Biosciences, #DF9044, Rabbit, 1:100), CD68 (Abcam,ab955,1:3000), CD206 (Cell Signaling,#24595,1:200), and CD163 (Cell Signaling,#93498,1:250) at 4°C overnight. Next, the sections were incubated with a biotinylated goat anti-rabbit IgG secondary antibody for 20 min at room temperature and visualized with 3, 5- diaminobenzidine (DAB) Substrate Kit and finally counterstained with Hematoxylin. The staining intensity was scored using a semi-quantitative approach as follows: 0, negative; 1, weak; 2, moderate; and 3, strong. The frequency of positive cells was defined as follows: 0, less than 5%; 1, 5–25%; 2, 26–50%; 3, 51–75%; and 4, greater than 75%. The final IHC scores were obtained by multiplying the staining intensity and the frequency of positive cells. When tissue staining was heterogeneous, each area was scored independently and the scores of each area were added together as the final result. Patient informed consents were obtained and approval of the internal review and ethics boards of the Affiliated Cancer Hospital and Institute of Guangzhou Medical University was also acquired.

### Cell lines and cell culture

Human gastric cancer cell lines (human gastric adenocarcinoma AGS & MNK45 cell lines) were obtained from Committee of Type Culture Collection of Chinese Academy of Sciences (Shanghai, China). Cells were grown in DMEM medium supplemented with 10% fetal bovine serum (FBS), penicillin (100 U/ml), and streptomycin (100 mg/ml). Cells were maintained at 37°C in a humid incubator (37°C, 5% CO_2_).

### Necroptosis induction

Necroptosis was induced in cells by treating them with a combination of recombinant human tumor necrosis factor-α (TNF-α) (Peprotech, New Jersey, USA; 10 ng/ml), second mitochondrial- derived activator of caspases (SMAC) mimetic (BV6; Selleck Chemicals, Houston, USA; 1 nM) and pan-caspase inhibitor (zVAD-FMK; ENZO Life Science, New York, USA; 40 μM). Necrostatin-1 (Enzo; 30 μM) was added an hour before treating with the above agents to inhibit necroptosis. To collect culture media, the cells were washed twice with PBS and media was replaced with fresh media after being treated for 3 hours with the aforementioned agents, which was followed by 12 hours’ incubation at 37 °C. The culture media (CM) was then collected and filtered with a 22-μm syringe filter (Merck, Darmstadt, Germany). Supernatants were collected after centrifugation at 1500 rpm for 5 minutes and stored at 4°C.

### Quantitative real-time PCR

Total RNA was extracted and purified using Trizol Reagent (Takara, Otsu, Japan) and it was reverse transcribed to cDNA and qRT-PCR were conducted by using a SYBR Green PCR Kit (Takara, Otsu, Japan). The expression of mRNA was standardized by internal control Glyceraldehyde 3-phosphate dehydrogenase (GAPDH), and relative mRNA level of the treated group was based on the control group. The Primers used in the study are presented in **Supplementary Table 3** (**Supplementary Data-Sheet#3**).

### Drug sensitivity

To predict the therapeutic vulnerability of high-risk group, the “pRRophetic” package in R was used to estimate the half-maximal inhibitory concentration (IC50) of drugs in the STAD patients (64). The pRRophetic algorithm uses the gene expression and drug sensitivity data from the cancer cell lines in the Cancer Genome Project (CGP) (65). The CGP investigated the therapeutic sensitivity of 130 drugs in more 639 cancer cell lines. The therapeutic targets, primary functions, or cellular functions of the 130 drugs included the following: serine/threonine kinase, receptor tyrosine kinase, cytoplasmic tyrosine kinase, cytoskeleton, metabolism, apoptosis, mitosis, replication, cell cycle, DNA repair, stress pathways, adhesion, transcription, angiogenesis, and chromatin. The panel of 130 encompassed 114 targeted and 13 cytotoxic chemotherapeutic agents. Of these, 31 are clinically approved, 47 in development undergoing clinical trials, and 52 were experimental tool compounds.

### Statistical analysis

Comparison of the gene expression level and drug sensitivity between the groups was accomplished using Wilcoxon test. Chi-square test was used to compare the categorical variables. Overall survival difference between the groups were estimated using the Kaplan-Meier method with log-rank test. Univariate and multivariate factor analyses were carried with cox-regression hazard models. All statistical analyses were performed with R software (v4.0.2).

## Results

### Differential expression of necroptosis-related genes between normal and tumor tissues

**Figure 1:**
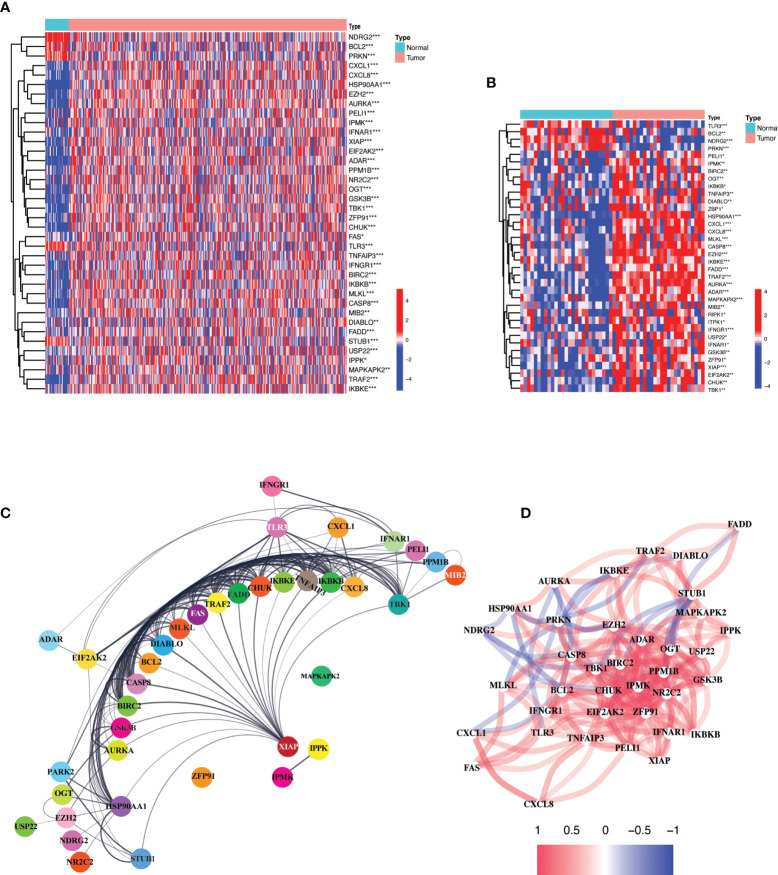
Expression and interaction of 55 necroptosis-related genes. **(A)** Heatmap of the NRGs between TCGA STAD tumor (n=375) and normal samples (n = 32). Red and blue represent upregulation and downregulation respectively. P values are shown as: *P < 0.05; **P < 0.01; ***P < 0.001. **(B)** Heatmap of NRGs between TCGA STAD paired normal (n = 32) and tumor samples (n = 32). Red and blue represent upregulation and downregulation respectively. P values are shown as: *P < 0.05; **P < 0.01; ***P < 0.001. **(C)** Protein-protein interaction (PPI) network demonstrating the interaction of NRGs (interaction score = 0.4). The interactions include direct (physical) and indirect (functional) associations; they stem from computational prediction, from knowledge transfer between organisms, and from interactions aggregated from other (primary) databases. **(D)** The correlation network of the NRGs based on mRNA expression in the TCGA STAD cohort. Red and blue lines indicate positive and negative correlation respectively. Color depth depicts the strength of the correlation.

Comparison of expression levels of the 55 necroptosis-related genes (NRGs) between 375 gastric cancer and 32 paired normal tissues obtained from the “The Cancer Genome Atlas (TCGA)” revealed 38 differentially expressed genes (DEGs) (all P < 0.01). Majority of the NRGs were deregulated in cancer tissues as compared to normal tissues which showed a uniform downregulated expression pattern except for NDRG2, BCL2, PRKN, TLR3, and STUB1. The mRNA levels of these genes are presented as heatmap in **Figure 1A**. A similar outlook was obtained when paired samples (n=32) from TCGA STAD cohort were considered only (**Figure 1B**). A protein-protein interaction was assessed for further exploration revealing a strong interaction activity among these molecules at protein level as demonstrated in **Figure 1C**. Likewise, the correlation network constructed based on the mRNA expression level in TCGA STAD demonstrated negative (blue) and positive (red) correlation among these NRGs as shown in **Figure 1D**. A strong positive correlation can be observed between majority of these NRGs. *These results indicate a critical deregulation of necroptosis in gastric cancer*.

### Identification of molecular subtypes

**Figure 2:**
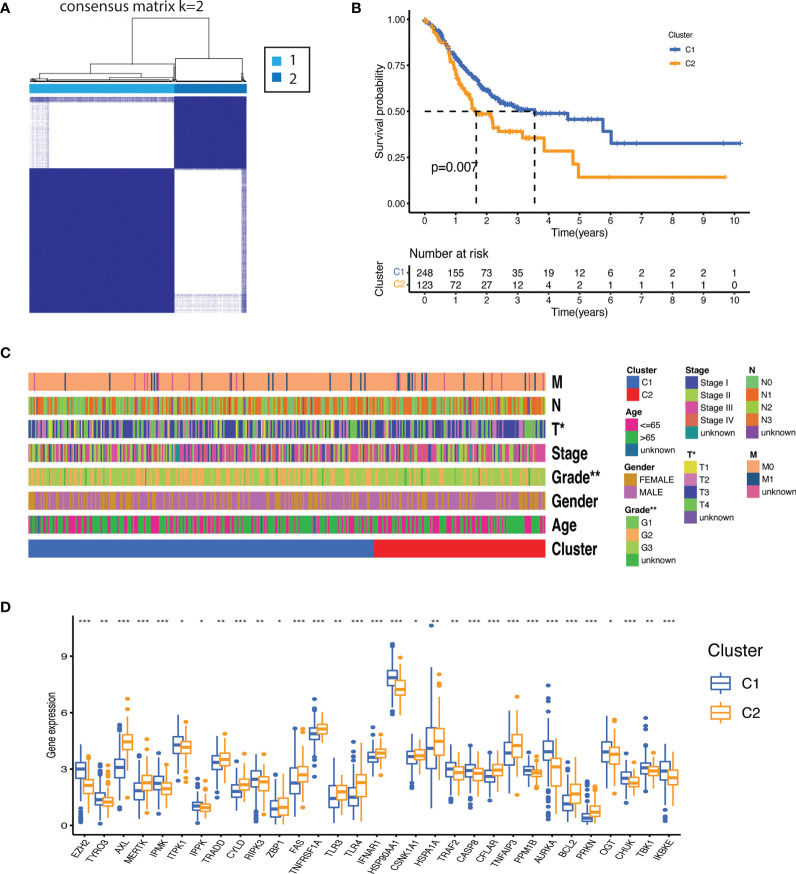
Molecular subtypes based on the necroptosis-related genes expression. **(A)** Consensus clustering matrix (k=2) identified two clusters (C1 = 248; C2 = 123) based on the expression of the 55 NRGs. **(B)** The clusters demonstrated significant difference in overall survival (p<0.007). **(C)** Heatmap illustrating association between the clusters and their clinicopathological features of the gastric cancer patients (TNM staging. T: primary tumor; N: lymph node; M: metastasis. Degree of differentiation. G1: highly differentiated; G2: moderately differentiated; G3: poorly differentiated). P value are shown as *P < 0.05; **P < 0.01. **(D)** Distribution of NRGs expression between the clusters (P values are shown as: *P < 0.05; **P < 0.01; ***P < 0.001).

Molecular subtypes were identified by subjecting necroptosis-related genes expression to consensus clustering. The 375 gastric patients were divided into two clusters (Cluster 1[C1] = 248 and Cluster 2[C2] = 123) as the intragroup correlations were the highest and the inter-group correlations were low when clustering variable (k) was equal to 2 (**Figure 2A**). The overall survival was significantly better for C1 as compared to C2 (p=0.007) (**Figure 2B**). Significant differences were also observed for clinical features such as tumor grade (degree of differentiation) and T stage (primary tumor) as highlighted in the **Figure 2C** and **Supplementary Table 4** (**Supplementary Data-Sheet#4**). We further sought the distribution of NRGs between the clusters. As shown in **Figure 2D**, cluster 1 was mainly characterized by the upregulation of RIPK3, inositol phosphates (IPMK, ITPK1, IPPK) and enhanced expression of negative regulators such as PPMIB, AURKA, OGT, TBK1, IKKα/β, IKKε, and TRAF2. While a stronger activity of TAM kinases (AXL and MERTK) and the main pathways receptors such as TNFR1, TLR3, TLR4, and FAS was demonstrated in the cluster 2. There was no significant difference between the clusters for RIPK1 and MLKL which indicates that both clusters may have undergone necroptosis *via* distinctive regulatory mechanisms which might have prompted differential negative regulation and prognosis.

### Development and validation of prognostic gene model

**Figure 3:**
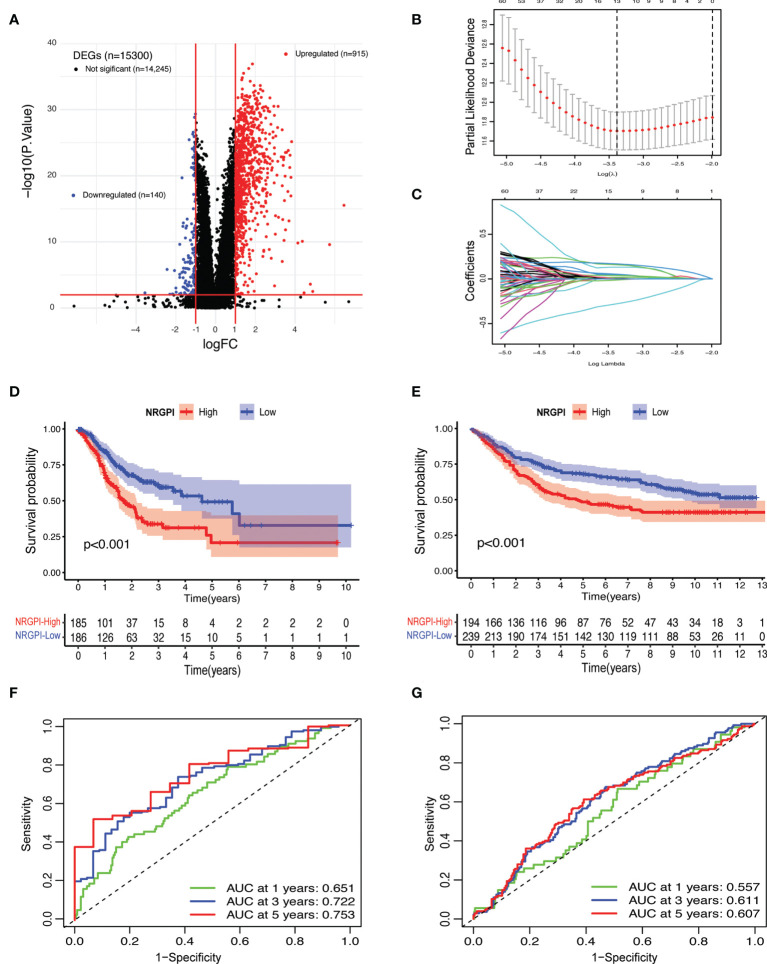
Risk signature construction. **(A)** Volcano plot depicting the differentially expressed genes (DEGs) between the necroptosis-based clusters. DEGs. Were defined according to the following criteria: log fold change (logFC) = 1, and the false discover rate (FDR) < 0.01. **(B)** LASSO regression of the 123 OS-related genes identified *via* uni-cox regression analysis. **(C)** Cross-validation for tuning the parameter selection in the LASSO regression. **(D)** Kaplan–Meier curves for the OS of NRGPI-High and NRGPI-Low patients in the TCGA cohort and **(E)** GEO cohort. **(F)** Time-dependent receiver operating characteristic (ROC) curves and area under curve (AUC) analyses depicting the predictive efficiency of risk score in TCGA cohort and **(G)** GEO cohort.

To evaluate the differences between the clusters, differential gene expression analysis was carried out which yielded 1055 differentially expressed genes (DEGs) according to the criteria (log fold change (logFC) = 1, and the false discover rate (FDR) < 0.01) (**Figure 3A** and **Supplementary Data-Sheet#5: Supplementary Table 5**). Both TCGA and GEO cohorts were screened for these DEGs (n=1055) and only gene expression of shared DEGs (n=860) was retained. After incorporation of survival information, both cohorts were subjected to prognostic analysis. Univariate cox regression analysis identified a total of 123 genes that showed a significant correlation with OS (**Supplementary Data-Sheet#6: Supplementary Table 6**). Among 123 survival genes, 119 survival genes were associated with increased risk (HR>1) and only four genes were protective genes with HR<1. Next, least absolute shrinkage and selection operator (LASSO) cox regression analysis was performed and a 13-gene risk signature, termed as necroptosis-related genes prognostic index (NRGPI) was obtained according to the optimum lambda (λ) value (**Figures 3B, C**). The risk score was calculated as follows: risk score = (0.0461 * CYTL1 expression) + (0.1313 * PLCL1 expression) + (0.1589 * CGB5 expression) + (0.0534 * CNTN1 expression) + (0.0104 * GRP expression) + (-0.0128 * EFNA3 expression) + (-0.0012 * E2F2 expression) + 0.0383 * APOD expression) + (-0.2798 * SOX14 ;expression) + (0.0044 * CST6 expression) + (0.0168 * GPX3 expression) + (0.0019 * FCN1 expression) + (0.1688 * SERPINE1 expression). Based on the median value of the risk score, samples were rated as low and high risk. A K-M plot, as depicted in **Figures 3D, E**, showed significantly worst survival for high-risk patients (NRGPI-High) versus low-risk patients (NRGPI-Low) in both TCGA and GEO cohorts (p<0.001). Diagnostic value of the prognostic model was evaluated with time-dependent receiver operating characteristic (ROC) analysis. The area under the curves (AUCs) were 0.651/0.722/0.753 at 1/3/5 years in the TCGA cohort, and 0.557/0.611/0.607 at 1/3/5 years in the GEO cohort (**Figures 3F, G**). Plotting of the risk scores indicated an equal distribution of patients into low- and high-risk groups (**Supplementary Figures 1A, B**). Patients in the NRGPI-High subgroup experienced more deaths and a shorter survival time (negative correlation) than those in the NRGPI-Low subgroup as demonstrated in **Supplementary Figures 1C, D**. Overall, a negative correlation was evident between survival time and risk score for both cohorts (**Supplementary Figures 1E, F**). Similarly, principal component analysis (PCA) and t-distributed stochastic neighbor embedding (t-SNE) showed well-separated clusters for the two risk groups (**Supplementary Figures 1G–J**).

### Independent prognostic assessment of the risk model

**Figure 4:**
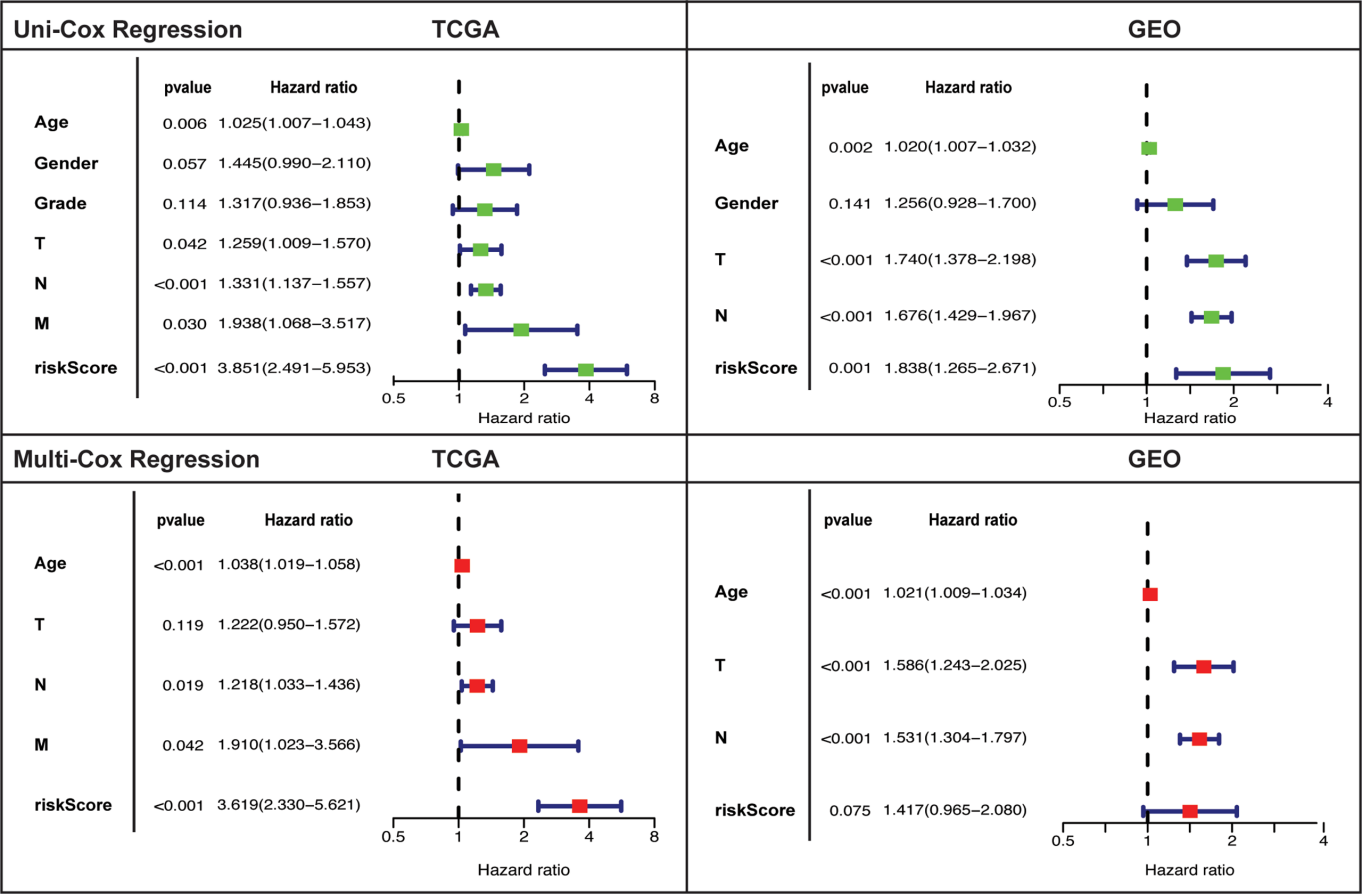
Univariate and multivariate cox-regression analysis to evaluate the independent prognostic value of the risk score in TCGA and GEO cohorts.

Independent prognostic value of the risk model and assessment of other clinical features as independent prognostic factors was evaluated by carrying out uni- and multi-variate cox regression analyses. Risk score was established as independent prognostic factor on univariate cox regression analysis in both cohorts (**Figure 4**). Risk score prognostic value was remained significant (only in TCGA and tended towards significance in GEO) after adjusting for confounding factors by undertaking multivariate analysis. Age and tumor stage also showed independent prognostic value in TCGA (univariate: T, N, and M; multivariate: N, and M) and GEO cohort (uni & multivariate: T and N).

### Risk model clinical and mutational evaluation

**Figure 5:**
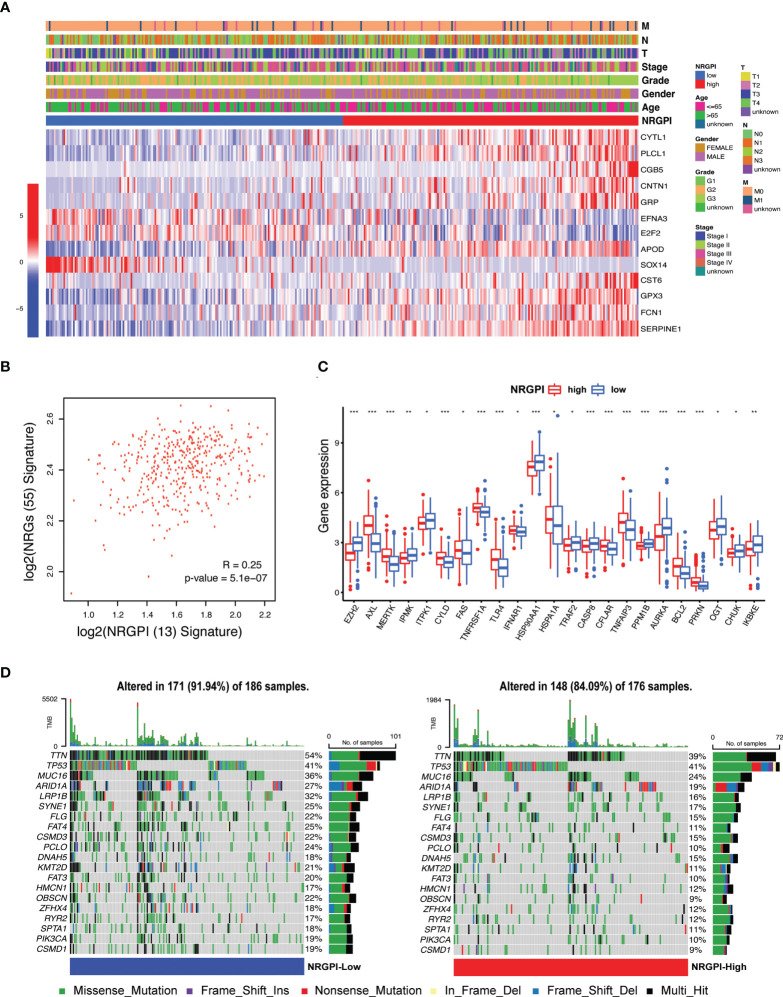
Expression and distribution of the risk signature genes and their correlation with clinical features. **(A)** Heatmap illustrating the expression of 13 risk genes (Red: upregulation; Blue: downregulation) in the NRGPI subgroups (Red: high-risk; Blue: low-risk) and correlation between NRGPI subgroups and clinicopathological features (TNM staging. T: primary tumor; N: lymph node; M: metastasis. Degree of differentiation. G1: highly differentiated; G2: moderately differentiated; G3: poorly differentiated). **(B)** Spearman’s correlation between 55 NRGs and NRGPI (13 risk genes) signature. **(C)** The expression levels of necroptosis-related genes between NRGPI-High and NRGPI-Low subgroups in the TCGA cohort (P values are shown as: *P < 0.05; **P < 0.01; ***P < 0.001). **(D)** Oncoplot depicting the mutation frequency of top 20 mutated genes in the high- and low-risk groups.

The expression level of the 13 risk genes and its correlation with clinical features are illustrated in heatmap (**Figure 5A**). No significant differences between NRGPI-risk subgroups for the clinical features were observed (**Supplementary Data-Sheet#7: Supplementary Table 7**). Of the 13 risk genes, expression of 10 genes (CYTL1, PLCL1, CGB5, CNTN1, GRP, APOD, CST6, GPX3, FCN1, SERPINE1) was unregulated in NRGPI-High subgroup. Overall, a positive correlation was evident between the 55 NRGs and 13 risk genes (**Figure 5B** and **Supplementary Figure 2A**). Significant differences in the 23 NRGs described the NRGPI-risk groups as compared to the clusters (significant difference in 31 NRGs between the clusters) (**Figure 5C**). Expression level of NRGs in the NRGPI-risk subgroups mirrored their expression status in the prognostic clusters. NRGPI-High subgroup showed significant elevated expression of TAM kinases (AXL and MERTK) and pathways receptors (TNFR1, FAS, and TLR4) as observed in the cluster 2. Likewise, inositol phosphates (IPMK, ITPK1) and negative regulators (PPMIB, AURKA, OGT, IKKε, and TRAF2) were expressed NRGPI-Low subgroup. These results indicate differential regulation of necroptosis in stomach adenocarcinoma. Moreover, mutations were less frequent in the NRGPI-High subgroup (91.94%) as compared to the NRGPI-Low subgroup (84.09%) (**Figure 5D**). Except for the TP53 gene, all the top 20 mutated genes showed lower frequency (up to 50% decrease) in the NRGPI-High subgroup in comparison to NRGPI-Low subgroup. Mutation frequency of the TP53 gene (second most mutated gene in gastric cancer) showed no difference at all between NRGPI subgroups and constituted the top mutated gene in the NRGPI-High subgroup (41%).

### Risk model functional implications

To evaluate functional implications of risk model, DEGs between the NRGPI subgroups defined by the risk model was obtained for evaluating differences between gene functions and pathways. The “limma” package was utilized with defined criteria: FDR < 0.05 and |log2FC | ≥ 1. According to this criterion, a total of 118 DEGs were identified between the NRGPI-High and NRGPI-Low subgroups in the TCGA cohort (**Supplementary Data-Sheet#8: Supplementary Table 8**). These DEGs were then subjected to Gene ontology (GO) enrichment analysis and Kyoto Encyclopedia of Genes and Genomes (KEGG) pathway analysis. Main biological process identified included extracellular matrix (ECM) structural organization, ECM-receptor interaction, cell-substrate adhesion and its regulation, negative regulation of cell motility and cellular component movement (**Supplementary Figures 2B, C**). Angiogenesis-related processes and pathways were also detected such as regulation of angiogenesis, vascular smooth muscle contraction, and cGMP-PKG signaling pathway. DEGs were also associated with complement and coagulation cascades, and proteoglycans in cancer. Moreover, signaling pathways implicated in cancer progression and immune suppression such as Wnt signaling pathway and TGF-β signaling pathway were also correlated.

### Immunological significance

**Figure 6:**
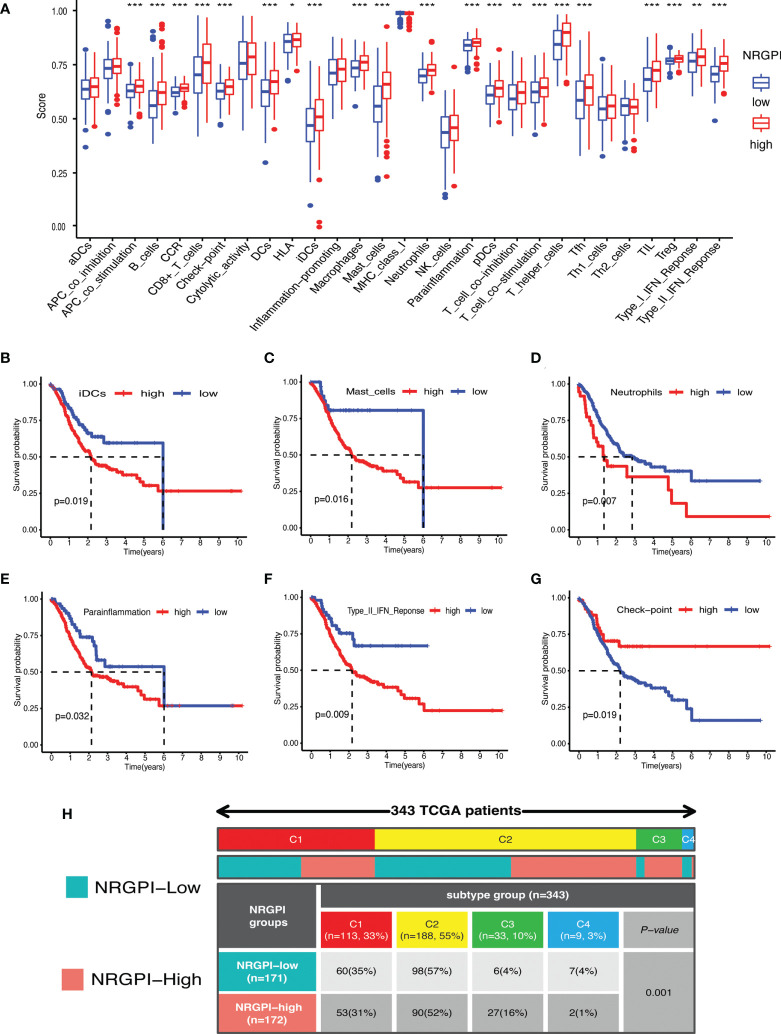
Immune landscape of NRGPI subgroups based on the single sample gene set enrichment analysis (ssGSEA) scores. **(A)** Comparison of enrichment scores of 16 types of immune cells and 13 immune-related pathways in the TCGA cohort between NRGPI-High and NRGPI-Low subgroups. **(B-D)** Kaplan-Meier curves for survival difference between TCGA patients with high and low- infiltration of immune cells. **(E-G)** Kaplan-Meier curves for survival difference between TCGA patients with high and low-activation of immune-related pathways. **(H)** Heatmap and table showing the distribution of immune subtypes (IC1, IC2, IC3, IC4, IC5, and IC6) between the NRGPI-High and NRGPI-Low subgroups. P values are shown as: *P < 0.05; **P < 0.01; ***P < 0.001.

**Figure 7:**
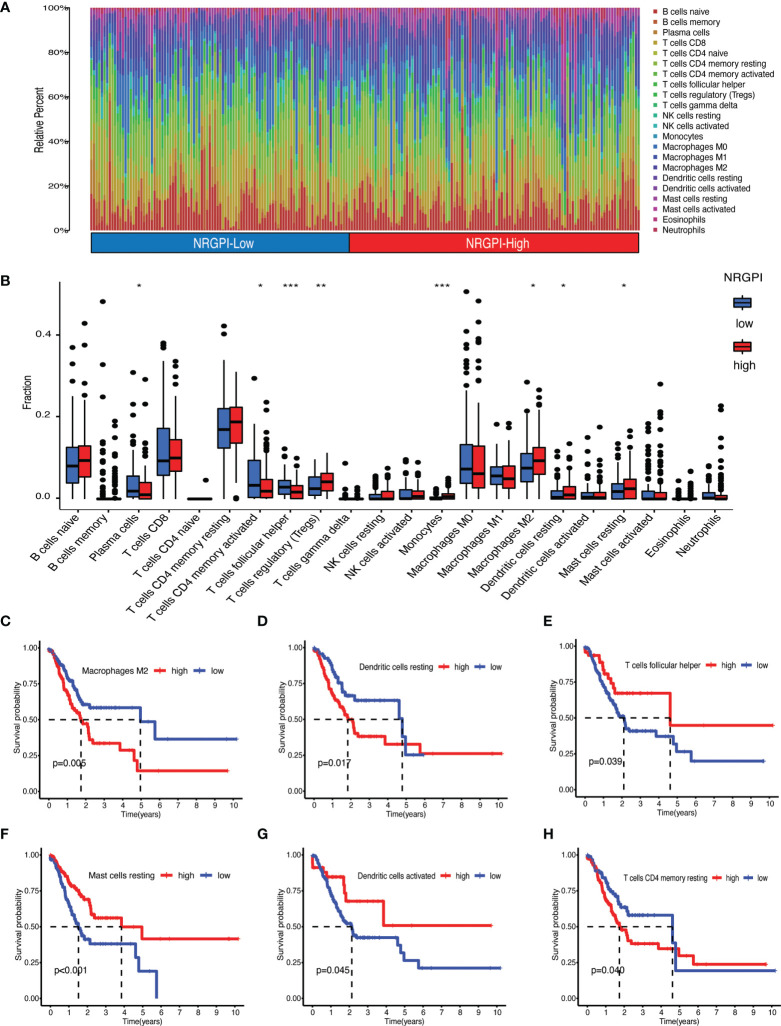
Immune landscape assessed by CIBERSORT algorithm. **(A)** Heatmap and **(B)** bar plot of abundance of 22 subtypes of immune cells in NRGPI-High and NRGPI-Low subgroups (P values are shown as: *P < 0.05; **P < 0.01; ***P < 0.001). **(C–H)** Kaplan-Meier curves for survival difference between TCGA patients with high and low- infiltration of immune cells.

Immunological significance was sought by undertaking immune enrichment analysis in terms of immune cells and immune-related pathways for the differences between NRGPI subgroups. Results of the single-sample gene set enrichment analysis (ssGSEA) involving TCGA and GEO cohorts indicated higher infiltration of various immune cells including B cells, dendritic cells (DCs), macrophages, mast cells, neutrophils, T helper cells, tumor infiltrating lymphocytes (TIL), and regulatory T cells (Treg) in NRGPI-High subgroup as compared to the NRGPI-Low subgroup (**Figure 6A** and **Supplementary Figure 3A**). Survival analysis based on the infiltration of immune cells indicated that a higher infiltration of immature dendritic cells (iDCs), mast cells, and neutrophils was associated with a worst prognosis (**Figures 6B–D**). All these three cells were more abundant in the NRGPI-High subgroup. To further dissect the various subtypes of immune cells, the CIBERSORT algorithm was used (**Figures 7A–H**). Results revealed that mainly M2 phenotype of the macrophages was more abundant in NRGPI-High subgroup and their infiltration was associated with a worst prognosis (**Figures 7B, C**). Resting DCs were also significantly abundant in the NRGPI-High subgroup, which was also associated with a worst outcome (**Figures 7B, D**). Likewise, different types of mast cells (resting and activated) also had prognostic significance (**Figures 7B, F**).

Immune-related pathways that were significantly upregulated in NRGPI-High subgroup (investigated in the TCGA cohort and validated in the GEO dataset) included APC co-stimulation, Chemokine receptors (CCR), checkpoint, para-inflammation, T cell co-stimulation, and type-II interferon (IFN) response. Upregulation of para-inflammation and type-II interferon pathways were associated with a worst prognosis (**Figures 6E, F**). On the other hand, checkpoint and T cell co-inhibition pathways, that were also slightly activated in the NRGPI-High subgroup, were associated with a better prognosis (**Figure 6G** and **Supplementary Figure 3F**). Other pathways and immune cells that had no significant differences between the cohorts had also significant impact on the prognosis (**Figure 6G** and **Supplementary Figures 3B-E, G**). Moreover, immune subtype analysis indicated that the main difference between the NRGPI subgroups was the comparative enrichment of inflammatory subtype in the NRGPI-High subgroup (C3: 6(4%) versus 27(16%, p=0.001) (**Figure 6H**).

### Construction of a ceRNA network

**Figure 8:**
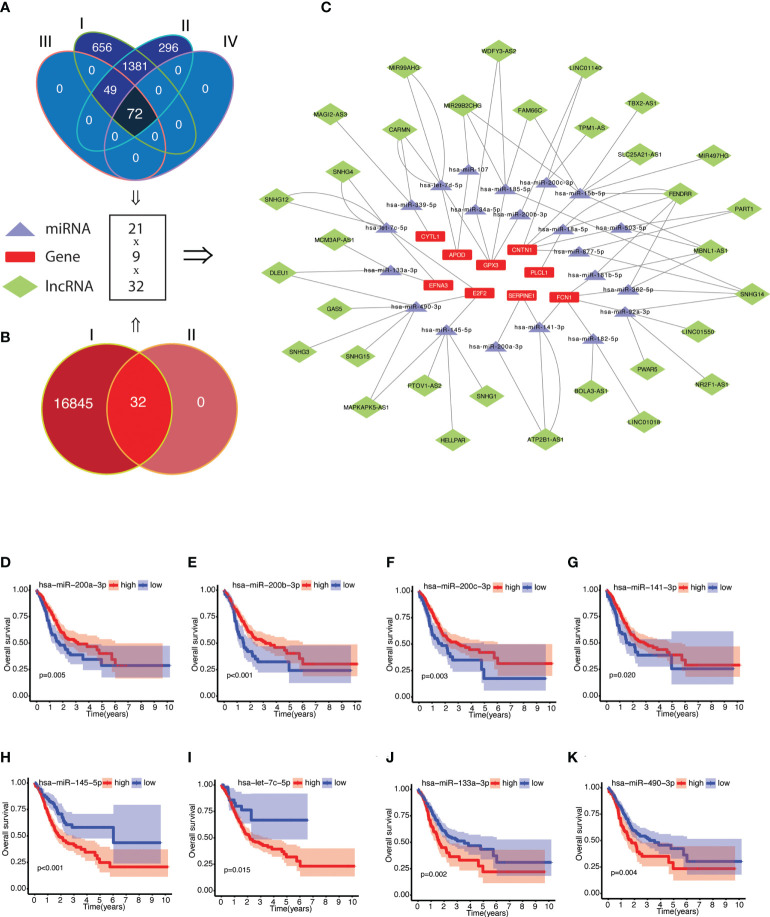
Construction of competing endogenous RNA (ceRNA) network of NRGPI genes. **(A)** Scrutiny of miRNA by (I) obtaining miRNA targets from miRWalk version 3.0 (n = 2158); (II) screening for miRNA expression in TCGA STAD cohort (n = 1798); (III) followed by gene-miRNA target correlation analysis (n = 122); and (IV) survival significance (n = 72). **(B)** lncRNA targets (n = 16877) were obtained for the 72 miRNA targets (I), which were then (II) screened for individual target-based correlation analysis (positive correlation with risk gene and negative correlation with miRNA). **(C)** A mRNA-miRNA-lncRNA (ceRNA) network (9 risk genes-21 miRNAs-32 lncRNAs) was constructed. Kaplan-Meier curves of survival analysis for miRNA targets of NRGPI oncogene **(D-G)** and NRGPI tumor suppressor genes **(H–K)**. Correlation was tested with Spearman’s correlation test with the following criteria: R=0.2 and p value <0.001.

The potential molecular mechanism of NRGPI genes was elucidated by constructing the network of mRNA–miRNA–lncRNA interactions as illustrated in **Figures 8A–C** (**Supplementary Data-Sheet 9-12: Supplementary Tables 9–12**). A total of 72 miRNA targets with prognostic significance were identified after screening for negative correlation with individual NRGPI genes (**Supplementary Data-Sheet#9, 10: Supplementary Tables 9, 10**). LncRNA targets (obtained from starbase v2.0) were screened for positive correlation with NRGPI genes and negative correlation with miRNA targets, which yielded 32 lncRNAs regulating the expression of 21 miRNAs and 9 NRGPI genes (**Supplementary Data-Sheet#11, 12: Supplementary Tables 11, 12**). Main miRNA families (miR-200, miR-15/107, let-7) were identified as regulator of NRGPI genes. For example, miRNA-200 family members (hsa-miR-200a-3p, hsa-miR-200b-3p, hsa-miR-200c-3p, and hsa-miR-141-3p), which are reported as tumor-suppressive group of miRNAs with essential role in suppressing EMT, were downregulated miRNAs (among the targeting miRNAs) in patients with higher expression of CNTN1, GPX3, FCN1 and SERPINE1 (NRGPI-high) (66). Additionally, CNTN1 was also regulated by hsa-miR-15b-5p and hsa-miR-503-5p (members of the microRNA-15/107 family) (67). APOD was regulated by hsa-miR-107 (another member of microRNA-15/107 family) and hsa-let-7d-5p which belongs to the let-7 family (68). PLCL1 and CYTL1 were negatively regulated by hsa-miR-18a-5p and hsa-miR-339-5p and positively regulated by lncRNA FENDRR and MAGI2-AS3, respectively. FENDRR was the predominant lncRNA regulator significantly correlating with 5 microRNAs and 3 NRGPI oncogenes. The tumor suppressive genes of the NRGPI (suppressed in NRGPI-High subgroup), the E2F2 and EFNA3, were regulated by a group of 4 microRNAs (E2F2: hsa-miR-490-3p and hsa-miR-145-5p; EFNA3: hsa-let-7c-5p and hsa-miR-133a-3p) (**Figures 8H–K**). The higher expression of these four microRNAs were predictive of worst prognosis as opposed to the other microRNAs regulating the oncogenes of NRGPI (**Figures 8D–G** and **Supplementary Figure 4**).

### Individual NRGPI genes evaluation and experimental validation

**Figure 9:**
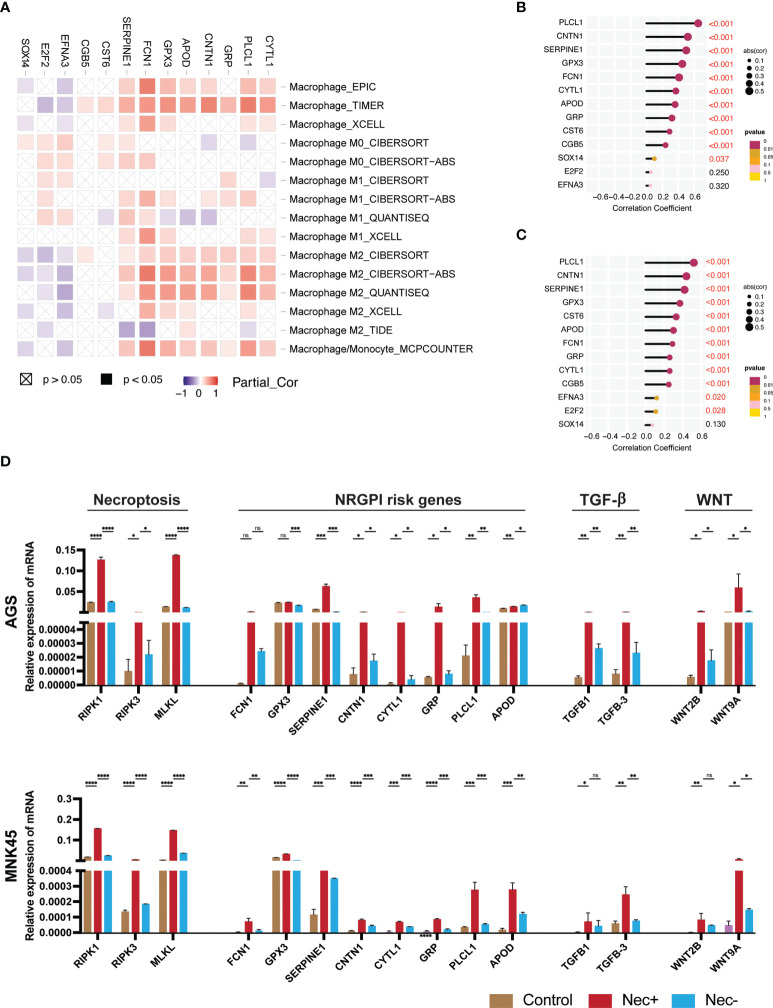
Individual assessment of NRGPI genes and association with necroptosis. **(A)** Spearman’s correlation between NRGPI genes and infiltration of macrophages in the TCGA STAD dataset assessed with TIMER database. **(B)** Spearman’s correlation between NRGPI genes and KEGG TGF-β signaling pathway signature genes and **(C)** WNT signaling pathway signature genes. **(D)** mRNA expression level of RIPK1, RIPK3, MLKL (necroptosis core mediators), NRGPI oncogenes (SERPINE1, GPX3, GRP, FCN1, CYTL1, CNTN1, PLCL1, and APOD), and markers of WNT signaling pathway (WNT2B, WNT9A) and TGF- signaling pathway (TGFB1, TGFB3) in gastric cancer cells (AGS and MNK45) after being treated with a combination of human recombinant TNF-α, SMAC mimetic, and zVAD-FMK (TSZ) to induce necroptosis or added necroptosis inhibitor (necrostatin-1) to inhibit necroptosis. Graph shows mean ± SD. *P<0.05; **P < 0.01; ***P < 0.001; ****P < 0.0001; ns, not signficant.

In order to experimentally validate the outcomes of our study, we further dissect the association of NRGPI genes with the characteristics of tumor microenvironment in gastric cancer such as wnt and TGF-β signaling pathways, and the infiltration of M2 macrophages. The individual NRGPI genes were evaluated for association with M2 macrophage in TCGA STAD cohort which revealed eight NRGPI oncogenes (SERPINE1, APOD, CYTL1, CNTN1, FCN1, GRP, GPX3, PLCL1) showed significant correlation with the infiltration of M2 macrophage (**Figure 9A**). In accordance with outcomes of GO enrichment analysis (**Supplementary Figure 2C**), NRGPI association with the two pathways was also investigated which showed strong correlation between the NRGPI and the two pathways (**Supplementary Figures 5A, B**). Interestingly, the aforementioned eight genes were among the most correlated with these pathways, in particular the TGF-β signaling pathway (**Figures 9B, C**). Hence, these eight NRGPI oncogenes were selected for further experimental analysis.

To confirm the association of necroptosis and NRGPI oncogenes, necroptosis was induced in gastric cancer cells (AGS and MNK45) using a combination of human recombinant TNF-α, SMAC mimetic, and zVAD-FMK (TSZ) as previously suggested (69, 70). After induction of necroptosis, the mRNA expression level of core mediators (RIPK1, RIPK3, and MLKL) were increased and suppressed when necroptosis inhibitor (necrostatin-1) was added (**Figure 9D**). There was statistical difference between the mRNA expression levels of these core mediators in both cell lines (AGS and MNK45). Moreover, a similar pattern of expression was evident for selected NRGPI oncogenes. The relationship between NRGPI and cancer progression pathways, such as WNT and TGF-β signaling pathways, was also confirmed as the mRNA expression level of WNT and TGF-β markers (wnt markers: WNT2B, WNT9A; TGF-β markers: TGFB1 and TGFB3), which were selected based on correlation analysis (**Supplementary Figures 5C, D**), were also significantly elevated with induction of necroptosis.

**Figure 10:**
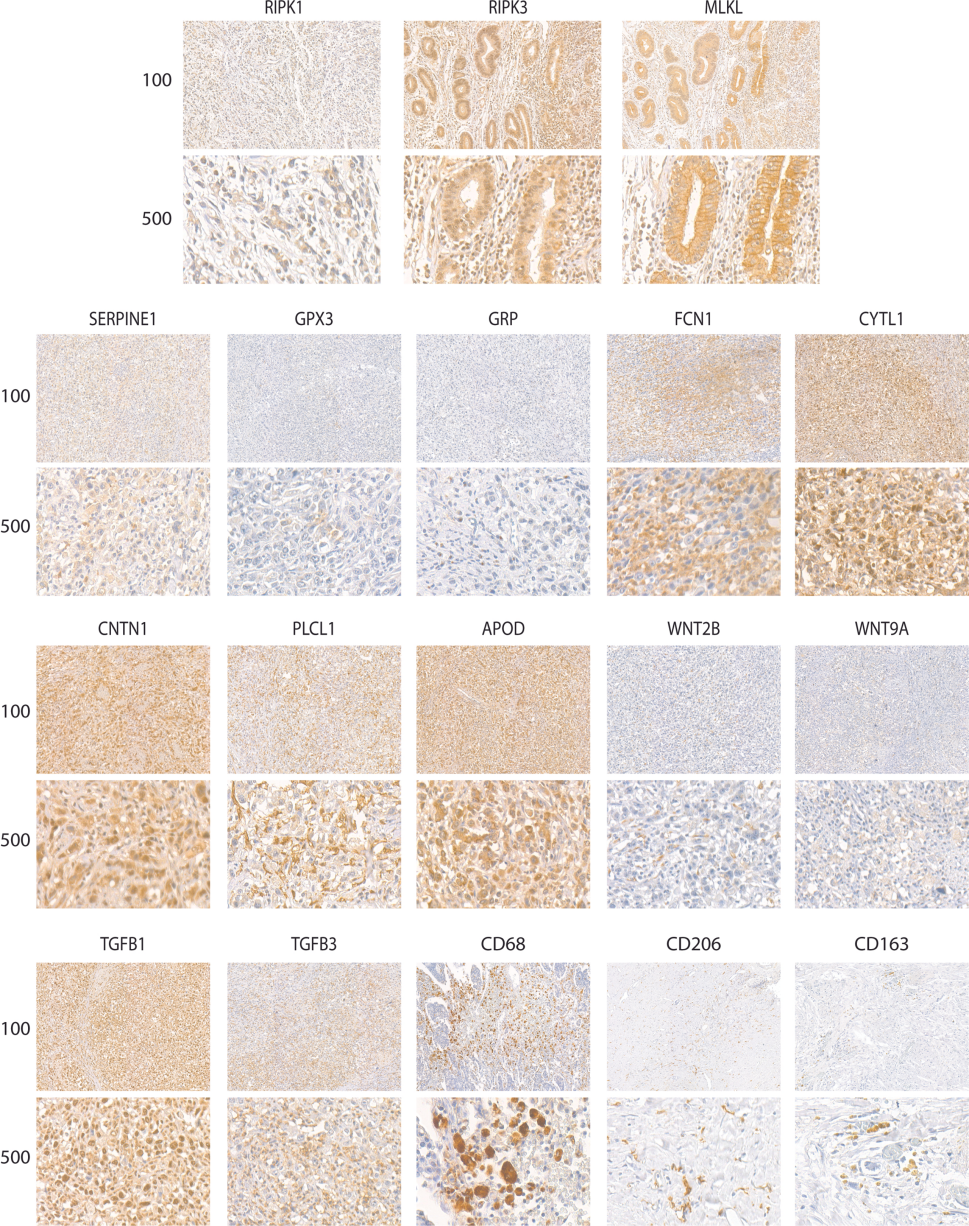
Representative images of expression (brown, cell cytoplasmic/nucleus stain) of RIPK1, RIPK3, MLKL (necroptosis core mediators), NRGPI oncogenes (SERPINE1, GPX3, GRP, FCN1, CYTL1, CNTN1, PLCL1, and APOD), markers of WNT signaling pathway (WNT2B, WNT9A), TGF-β signaling pathway (TGFB1, TGFB3), and macrophage (CD63, CD206, CD163) in the clinical samples of stomach adenocarcinoma.

**Figure 11:**
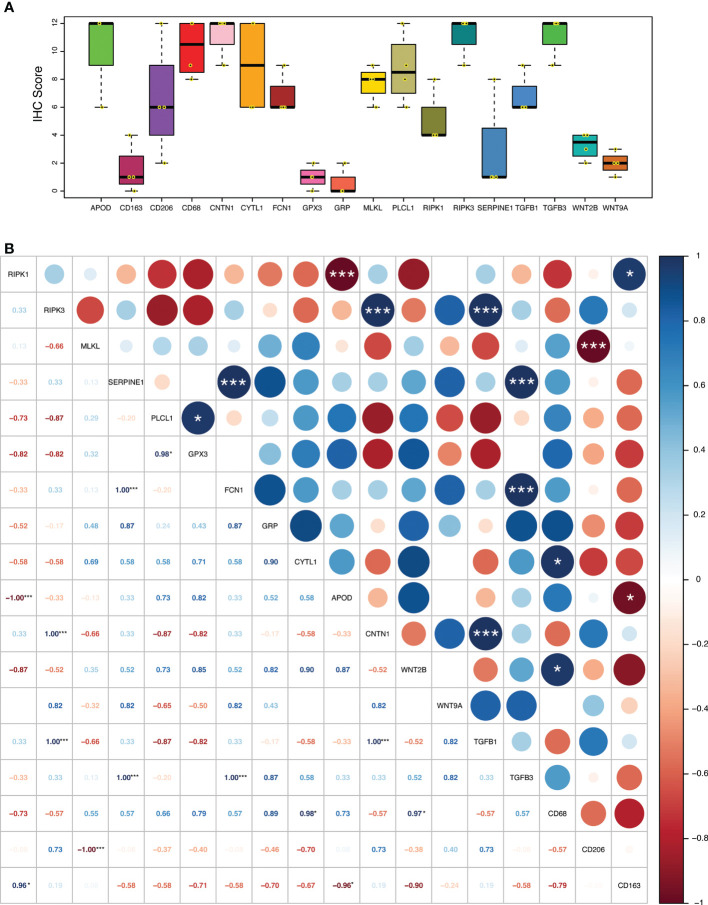
**(A)** Expression level (IHC quantification) of RIPK1, RIPK3, MLKL (necroptosis core mediators), NRGPI oncogenes (SERPINE1, GPX3, GRP, FCN1, CYTL1, CNTN1, PLCL1, and APOD), and markers of WNT signaling pathway (WNT2B, WNT9A), TGF-β signaling pathway (TGFB1, TGFB3), and macrophage (CD63, CD206, CD163) in the clinical samples (n = 4) of stomach adenocarcinoma. The scattered dots represent the IHC score of each individual sample. The thick middle lines represent the median value and error bars indicate the standard deviation. The bottom and top of the boxes are the 25th and 75th percentiles (interquartile range), respectively. **(B)** A correlation matrix illustrating Pearson’s correlation coefficients indicating the relationship among expression levels of RIPK1, RIPK3, MLKL (necroptosis core mediators), NRGPI oncogenes (SERPINE1, GPX3, GRP, FCN1, CYTL1, CNTN1, PLCL1, and APOD), and markers of WNT signaling pathway (WNT2B, WNT9A), TGF-β signaling pathway (TGFB1, TGFB3), and macrophage (CD63, CD206, CD163) in the clinical samples (n = 4) of stomach adenocarcinoma. P values are shown as: *P < 0.05; **P < 0.01; ***P < 0.001.

The relationship of necroptosis, NRGPI oncogenes and tumor microenvironment characteristics was further established in clinical samples *via* conducting immunohistochemistry analysis in stomach adenocarcinoma patients. As shown in **Figures 10**, **11A, B**, the core mediators of necroptosis (mainly RIPK3 and. MLKL) were highly expressed in these patients which correlated with several of NRGPI oncogenes such as APOD, CYTL1, CNTN1, and PLCL1. TGF-β pathway was evidently activated as the both of the receptors (TGFB1 and TGFB3) showed considerable expression in these patients (**Figure 11A**). Moreover, macrophage infiltration was intensely evident and showed a predominant positive correlation with the NRGPI oncogenes and pathway markers (**Figures 11A, B**). M2 phenotype expression (CD206 and CD163) was also demonstrated which, in general, showed a negative correlation but certain significant positive correlation was also apparent. For example, RIPK3 expression was significantly positively correlated with CNTN1 (p<0.001), TGFB1 (p<0.001), and CD206 (not significant). *Overall, these results indicate the presence of a strong relationship as suggested by bioinformatics outcomes between the necroptosis, NRGPI oncogenes, and the tumor immune microenvironment which should further be elaborated on individual level.*

### Therapeutic response

The drug sensitivity profile highlighted two main aspects of the NRGPI subgroups (**Supplementary Figure 6**). Of the 13 chemotherapy agents, 4 agents showed sensitivity to NRGPI-Low subgroup and none to NRGPI-High subgroup. Major tyrosine kinase inhibitors, that mainly target epidermal growth factor receptor (EGFR), phosphoinositide 3-kinases (PI3K), mammalian target of rapamycin (mTOR), SRC kinases, BCR-Abl, vascular endothelial growth factor receptor (VEGFR), and platelet-derived growth factor receptor (PDGFR), showed sensitivity to NRGPI-High subgroup. Moreover, two poly (ADP-ribose) polymerase (PARP) agents also showed lower IC50 values for the NRGPI-High subgroup. Interestingly, the NRGPI-High subgroup showed resistant to polo-like kinase 1 (PLK1) inhibitors. Detailed profile of the drug sensitivity is listed in **Supplementary Table 13** (**Supplementary Data-Sheet#13**).

## Discussion

**Figure 12:**
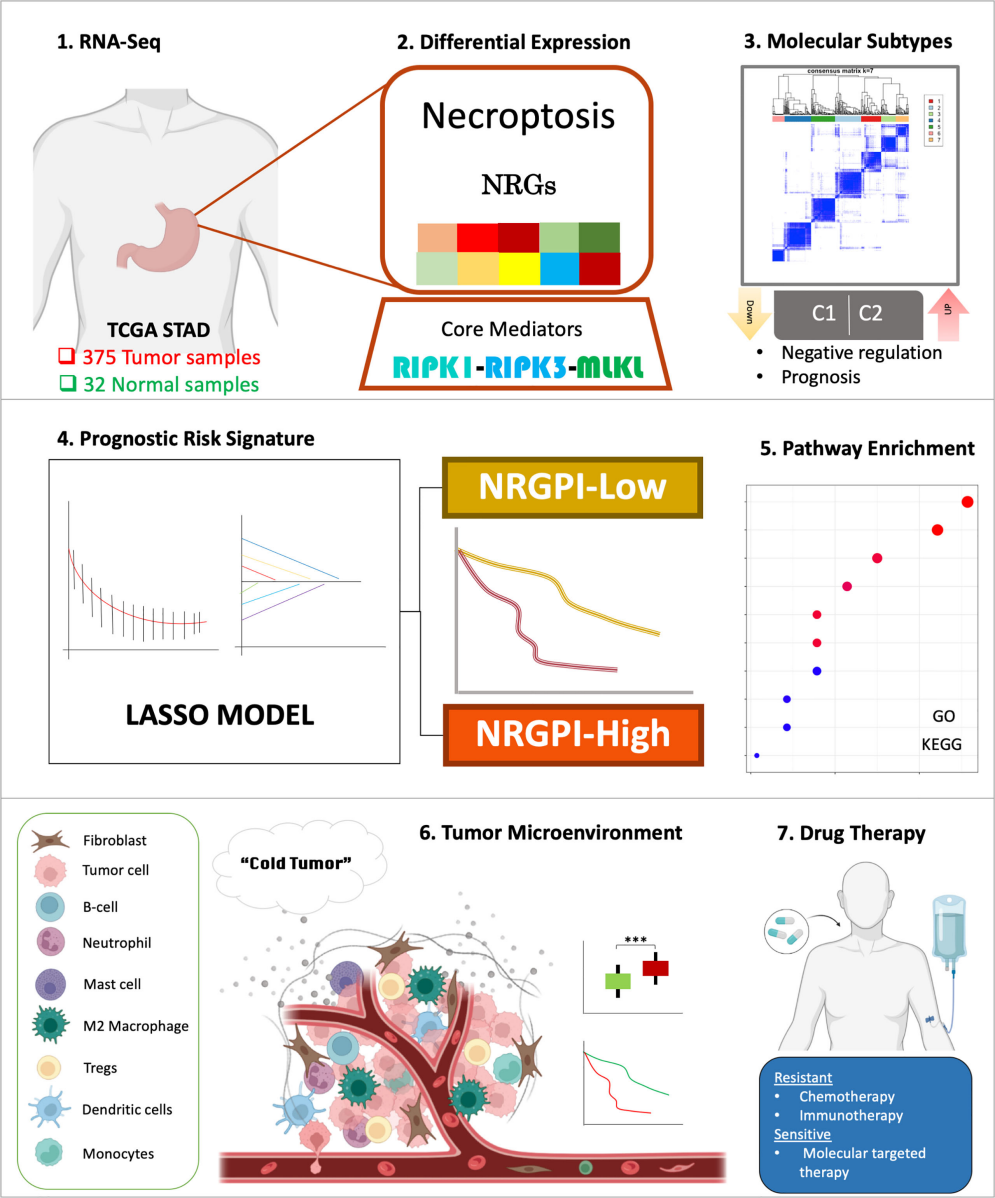
Graphical abstract of construction and characterization of NRGPI subgroups.

Necroptosis pathway was greatly deregulated in gastric cancer samples as compared to adjacent normal tissues, wherein the majority of the necroptosis-related genes (NRGs) demonstrated a uniform downregulation. Differential expression of NRGs also identified two molecular subtypes that showed significant difference in prognosis. A 13-gene risk signature was constructed based on the differential expression of genes between the molecular subtypes, that comprehensively differentiated the gastric cancer patients into high and low-risk subgroups. Dissection of these two risk groups by differential gene expression analysis indicated involvement of several biological processes related to cell motility, extracellular organization, and signaling pathways associated with cancer cell progression and immune suppression such as WNT signaling pathway and TGF-β signaling pathway. Para-inflammation and type-II interferon response pathways were evident with an increased infiltration of regulatory T cells (Tregs) and M2 macrophages. Overall, an exhausted immune phenotype was apparent in the NRGPI-High subgroup (**Figure 12**).

In general, current evidence is insufficient to conclude the role of necroptosis as pro-tumorigenic or anti-cancer process. Down-regulation of core mediators such as RIPK3 and MLKL indicates an attempt to escape from necroptosis. On the other hand, its role in promotion of metastasis and T cell death underlines its role in carcinogenesis. While its inflammatory nature and promotion of cross-priming holds potential for targeted therapy. The clustering based on NRGs indicated two patterns of expression for necroptosis that was also evident in the NRGPI subgroups. Cluster with the worst prognosis (C2 & NRGPI-High subgroup) was identified by the upregulation of necroptosis-associated receptor activity and TAM kinases, AXL and MERTK. TAM kinases promote necroptosis *via* regulating MLKL oligomerization (23). Although MLKL showed no differential expression between the NRGPI subgroups, it was the only differentially expressed core necroptotic mediator in cancer samples as compared to normal tissues. Both of these indicators plus the lower expression of negative regulators indicate this cohort may have undergone significant necroptosis. While the cluster with a better prognosis (C1 & NRGPI-Low subgroup) was enriched in the expression of negative regulators, RIPK3 upregulation and inositol phosphates, which may indicate higher suppression or resistance to necroptosis in this cohort. Like TAM kinases, inositol phosphates, IPMK, ITPK1, and IPPK, have also been reported for their role in execution of necroptosis *via* activation of MLKL (27, 28). This could also explain the lack of significant differential expression of MLKL between the cohorts. Hence, under different circumstances, the necroptosis process may or may not be suppressed or resisted which holds prognostic significance. Further investigations would be required to investigate the mechanistic details of this phenomenon.

In our study, we identified a 13-gene risk signature (NRGPI) which characterized the gastric cancer patients into a NRGPI-High- and NRGPI-Low patients based on the differential gene expression between the two clusters. The study of these genes may help to comprehend the mechanism of differential necroptotic status and outcome between the aforementioned clusters. The 13-gene risk signature included several genes that have previously been implicated in cancer development and progression. Cytokine-like 1 (CYTL1, also known as C17 or C4ORF4) is a secreted protein that has shown a deregulated expression profile across cancers and has also been implicated in carcinogenesis (71). A previous study has reported its role in chemoattraction of monocytes *via* the CCR2/ERK pathway, which were more abundant in the NRGPI-High subgroup compared to the NRGPI-Low subgroup (72). Moreover, CYTL1 expression was positively correlated with macrophage marker CD68 indicating an interplay between necroptosis (CYTL1 positively correlated with MLKL), CYTL1 and macrophage infiltration which needs further exploration. It was identified as a tumor suppressor in lung cancer *via* inhibiting the tumor invasion and metastasis rather proliferation (72). While a similar effect on tumor growth and metastasis was achieved *via* inhibition of metabolic reprogramming in breast cancer cells expressing an intracellular form of CYTL1 that lacked a 1-22 aa signal peptide, ΔCYTL1 (73). In contrast, CYTL1 was involved in the growth and metastasis of neuroblastoma cells (74). Although its expression is downregulated in gastric cancer, a higher expression suggested a poor prognosis as shown in our study. Furthermore, our study identified the hsa-miR-339-5p as miRNA target of CYTL1 which has previously been recognized as a suppressor of malignant development in gastric cancer thereby further cementing CYTL1 role as an oncogenic molecule in gastric cancer (75). Phospholipase C like 1 (PLCL1) is required for insulin induced gamma-aminobutyric acid type A (GABA(A)) receptor expression and in the gonadotropin secretion (76, 77). PLCL1 was demonstrated to induce abnormal lipid metabolism in tumor cells by interacting with metabolism-related gene uncoupling protein 1 (UCP1), thereby repressing progression of clear cell renal cell carcinoma (ccRCC) (78). In a breast cancer study, its expression was associated with PIK3CA mutation and PFS; nonetheless, its role in cancer has largely been unknown (79). Our study indicated PLCL1 to play a role in macrophage infiltration as shown in bioinformatic analysis and validated in the immunohistochemistry analysis of the clinical samples. In coherence with our study, chorionic gonadotropin subunit beta 5 (CGB5) – a protein-encoding gene primarily associated with invasive mole and ectopic pregnancy – has previously been identified as a biomarker in gastric cancer (80). It exhibits structural similarities with other growth factors and has been shown to act as proangiogenic factor in some tumors (81, 82). Other properties include suppression of apoptosis and induction of epithelial‐to‐mesenchymal transition (EMT) *via* TGF-β signaling pathway (83–85). These functional properties of CGB5 could also be reflected in our study.

Contactin 1 (CNTN1), a neuronal membrane glycoprotein, has long been implicated in cancer cell invasion, migration, metastasis *via* the epithelial-mesenchymal transition (EMT) in several cancers such as the lung cancer, gastric cancer, esophageal cancer, thyroid cancer, liver cancer, prostate cancer, and breast cancer (86–93). In fact, CNTN1 was identified as a critical NRGPI oncogene in our study which showed strong association with necroptosis (RIPK3), TGF-β signaling pathway (TGFB1), and infiltration of the M2 macrophages (CD206). Moreover, ceRNA network identified hsa-miR-200c-3p as regulator of CNTN1 which further assert CNTN1 role in gastric cancer development *via* EMT (66). Hence, CNTN1 may induce TGF-β1 which can promote differentiation of macrophages into M2 phenotype thereby further inducing the secretion of TGF-β1 and consequently prompting the EMT (94, 95). In has also been involved in the development of chemoresistance in lung cancer (96). Several studies have clearly indicated CNTN1 as an independent prognostic factor in gastric cancer 87, 88). Gastrin releasing peptide (GRP) is a neuropeptide that causes the secretion of gastrin in the stomach (97). GRP is over-expressed in a number of cancers including lung, breast, stomach, pancreas, renal, prostate, and colon (98, 99). GRP actions relevant to carcinogenesis include its role as a potent mitogen and its effects on angiogenesis, cell adhesion, and cell migration – pathways that were also revealed in our study (100, 101). Apolipoprotein D (ApoD), a protein regulated by androgen and estrogen, is implicated in breast cancer as a poor prognostic factor (102). In gastric cancer, several bioinformatic analysis have revealed APOD as a component of gene-risk model and associated with tumor mutational burden and immune cell infiltration (103–105). Our study further validated these characteristics of APOD demonstrating a positive correlation with infiltration of macrophages (CD68) and WNT signaling pathway (WNT2B). Cystatin E/M (CST6), a representative cysteine protease inhibitor, has been well appreciated as a tumor-promoting and tumor-suppressing agent and is pursued as an epigenetically therapeutic target in special cancer types (106). Loss of expression in 70% of gastric cancer was reported due to promoter hypermethylation which was associated with shorter survival (107). Moreover, CST6 was also part of a CpG island methylator phenotype-related prognostic gene signature which differentiated gastric cancer into high-and low-risk groups with a significant OS difference (108). Although the DNA methylation status of CST6 was not determined in our study, low expression of CST6 was associated with better prognosis. Likewise, extracellular glutathione peroxidase (GPX3) also plays a dichotomous role in different types of cancer (109). Bioinformatic analysis of TCGA data have revealed poor prognosis for gastric patients with higher GPX3 expression, which is in coherence with the outcome of our study. However, a tumor suppressive role also been reported for GPX3 in gastric cancer wherein its knockdown resulted in tumor cell invasion and migration by targeting NFкB/Wnt5a/JNK signaling (110). Ficolin-1 (FCN1) is a member of the ficolins family proteins that are considered as multifunctional innate immune defense factors mainly associated with complement pathway. Their role in cancer is not exclusively elaborated (111). Our study indicates association of higher expression of FCN1 with poor prognosis. Further exploration of these factors is warranted as a therapeutic target in gastric cancer. Serpin family E member 1 (SERPINE1) encodes plasminogen activator inhibitor 1 (PAI-1), which is a primary inhibitor of tissue plasminogen activator (tPA) (112). It has been detected in various cancer and involved in cancer invasion, migration, and angiogenesis (112–116). Activation of PAI-1 transcription is mediated by the cooperation of tumor suppressor p53 with TGF-β signal transducers, Smad proteins, to selectively enhance TGF-β-induced cytostatic effects (117). In gastric cancer, it was highly expressed and associated with regulation of EMT (114). Our study indicated PAI-1 as one of the NRGPI oncogenes associated with the TGF-β and infiltration of M2 macrophages.

Ephrin A3 (EFNA3), like most genes in the ephrin family, plays a central role in embryonic development and can be dysregulated in a variety of tumors (118). In gastric cancer, it has been identified as part of the prognostic gene signature during investigation of hypoxia and glycolysis (119, 120). Interestingly, the expression of Ephrin A3 was down-regulated in the high-risk group. Indicating it as tumor suppressive factor as reported before (121). E2Fs, transcription factor protein family, are implicated in carcinogenesis for their role in cell cycle control (122, 123). Previously, E2F2 has been reported for its role in development of gastric cancer growth (124). However, it was downregulated in the high-risk cohort indicating it as a tumor suppressive target. SOX14, a transcription factor, has largely been unexplored in cancer.

Investigations of immune-related pathways revealed activation of para-inflammation, a low-grade form of inflammation, in the high-risk group. Para-inflammation is implicated in the cancer development (125). Viral infection such as Epstein-Barr virus (EBV) and chronic infections such as H. Pylori might contribute to the para-inflammatory status (126). This status could certainly activate the innate immune pathways as observed in our study including APC co-stimulation, and type-II interferon secretions. Enrichment of TP53 mutation has also been linked to parainflammation-positive tumors, which was the highly mutated gene in the high-risk group (125). TP53 role in cancer cell cycle is mediated *via* p53-TGF-β signaling pathway which showed comparative enrichment in the high-risk group. Moreover, the immune landscape indicated a predominantly innate immune phenotype for the high-risk group, which was characterized by high infiltration of monocytes, M2 macrophages, activated mast cells, resting dendritic cells and regulatory T cells (Tregs). The survival analysis indicated significant impact on prognosis for the infiltration of these cells. Infiltration of Tregs as well as M2 macrophage phenotype have previously been associated with poor prognosis in cancers including gastric cancer (127–129). Overall, an exhaustive immune subtype is apparent for the high-risk patients characterized by the activation of Wnt and TGF-β pathways and the abundance of M2 macrophage and Tregs (130). As such, the high-risk patients may not respond well to the immune checkpoint inhibition therapy. Our investigations of the immune subtype identified the major difference between the cohorts which was the enrichment of inflammatory immune subtype in the high-risk group. Inflammatory subtype is characterized by the highest infiltration of Th17 cells among the immune subtypes, which is also implicated in cancer (61). TGF-β in the gastric tumor microenvironment is reported to promote the differentiation and expansion of both Th17 cells, Tregs and M2 macrophage (94, 95, 131, 132). Th17 contribute to gastric cancer growth through promotion of inflammation and secretion of IL-17 as opposed to Tregs which is involved in immune surveillance (132). Overall, the exhausted immune microenvironment may not be a suitable candidate for immunotherapy. Interestingly, resistance to certain chemotherapy agents was also apparent in our study. However, selective molecular targeted therapy, as demonstrated in the drug sensitivity analysis, might be a better option for the NRGPI-High gastric patients.

## Conclusions

Necroptosis appears to play a critical role in the development and progression of gastric cancer. Molecular subtypes could further dissect the differential role necroptosis might play during the gastric cancer progression with implications for tumor immune microenvironment, prognosis, and therapy. Results of our study could provide a basis for further work on elaborating the mechanistic details of necroptosis in gastric cancer.

## Data availability statement

The mRNA expression and clinical data used in this article can be accessed from TCGA (https://portal.gdc.cancer.gov/) and GEO (https://www.ncbi.nlm.nih.gov/geo/) databases. The accession number(s) can be found in the article/**Supplementary Material**.

## Ethics statement

The studies involving human participants were reviewed and approved by Internal review and ethics boards of the Affiliated Cancer Hospital and Institute of Guangzhou Medical University. The patients/participants provided their written informed consent to participate in this study.

## Author contributions

MK, JL, and BW contributed equally to this work. All authors have made significant contributions to the conception, supervision, and final approval of the manuscript.

## Funding

This study was supported by the National Natural Science Foundation of China (82102974), Science and Technology Program of Guangzhou, China (202201011048), and Key Clinical Technology of Guangzhou (2019ZD17).

## Conflict of interest

The authors declare that the research was conducted in the absence of any commercial or financial relationships that could be construed as a potential conflict of interest.

## Publisher’s note

All claims expressed in this article are solely those of the authors and do not necessarily represent those of their affiliated organizations, or those of the publisher, the editors and the reviewers. Any product that may be evaluated in this article, or claim that may be made by its manufacturer, is not guaranteed or endorsed by the publisher.

## Supplementary material

Supplementary Figure 1 Assessment of the risk model. **(A)** Distribution of TCGA and **(B)** GEO patients based on the risk score. **(C)** Risk scores and survival correlation for the high- (on the right side of the dotted line) and low-risk (on the left side of the dotted line) TCGA and **(D)** GEO patients. **(E)** Linear regression between risk score and survival time in years for TCGA and **(F)** GEO cohorts. **(G)** Principal component analysis (PCA) plots of risk scores for TCGA and H) GEO cohorts. **(I)** t-distributed stochastic neighbor embedding (*t-SNE*) plots of risk scores for TCGA and **(J)** GEO cohorts.

Supplementary Figure 2 Correlation and functional analysis of the risk groups. **(A)** Pearson’s correlation between individual NRGs (n = 55) and NRGPI (n = 13). P values are shown as: *P < 0.05; **P < 0.01; ***P < 0.001. **(B)** Circos plot depicting the enrichment of gene ontology (GO) terms (only biological process: BP) and **(C)** *Kyoto Encyclopedia of Genes and Genomes* (KEGG) pathways (increasing depth of the red indicate the more obvious differences; q-value: the adjusted p-value).

Supplementary Figure 3 Immune landscape of NRGPI subgroups based on the single sample gene set enrichment analysis (ssGSEA) scores. **(A)** Comparison of enrichment scores of 16 types of immune cells and 13 immune-related pathways in the GEO cohort between NRGPI-High and NRGPI-Low subgroups. **(B–F)** Kaplan-Meier curves for survival difference between TCGA patients with high and low-activation of immune-related pathways. **(G)** Kaplan-Meier curves for survival difference between TCGA patients with high and low- infiltration of Th2 cells. P values are shown as: *P < 0.05; **P < 0.01; ***P < 0.001.

Supplementary Figure 4 Kaplan-Meier curves of survival analysis for miRNA targets of NRGPI oncogenes.

Supplementary Figure 5 Association of NRGPI and cancer-associated pathways **(B)** Spearman’s correlation between NRGPI (13 risk genes) signature and KEGG TGF-β signaling pathway (86-gene signature) in TCGA STAD cohort. **(B)** Spearman’s correlation between NRGPI (13 risk genes) signature and KEGG WNT signaling pathway (151-gene signature) in TCGA STAD cohort. **(C)** Pearson’s correlation between individual NRGPI (n=13) and markers of TGF-β signaling pathway in TCGA STAD cohort. P values are shown as: *P < 0.05; **P < 0.01; ***P < 0.001. **(B)** Pearson’s correlation between individual NRGPI (n=13) and markers of WNT signaling pathway in TCGA STAD cohort. P values are shown as: *P < 0.05; **P < 0.01; ***P < 0.001.

Supplementary Figure 6 Prediction of drug sensitivities of NRGPI-High and NRGPI-Low subgroups in TCGA cohort.
