# Supplementary figures and images for: A novel necroptosis-related gene index for predicting prognosis and a cold tumor immune microenvironment in stomach adenocarcinoma
Source: Front Immunol. 2022 Oct 27;13:968165. doi: 10.3389/fimmu.2022.968165 (PMC9646549; doi:10.3389/fimmu.2022.968165)

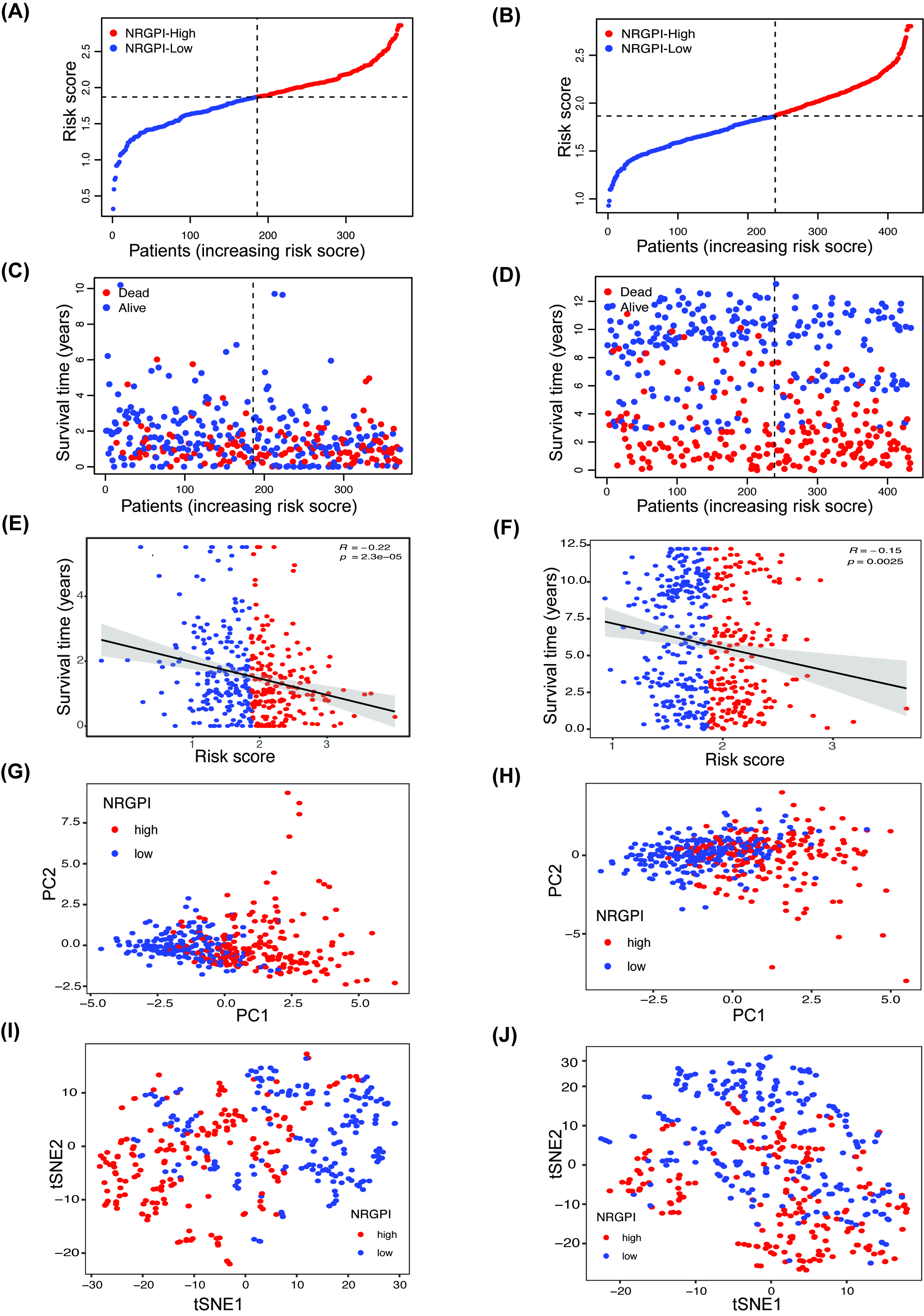

Supplement: Supplementary Figure 1 — Assessment of the risk model. (A) Distribution of TCGA and (B) GEO patients based on the risk score. (C) Risk scores and survival correlation for the high- (on the right side of the dotted line) and low-risk (on the left side of the dotted line) TCGA and (D) GEO patients. (E) Linear regression between risk score and survival time in years for TCGA and (F) GEO cohorts. (G) Principal component analysis (PCA) plots of risk scores for TCGA and H) GEO cohorts. (I) t-distributed stochastic neighbor embedding (t-SNE) plots of risk scores for TCGA and (J) GEO cohorts. [file Image_1.tif]

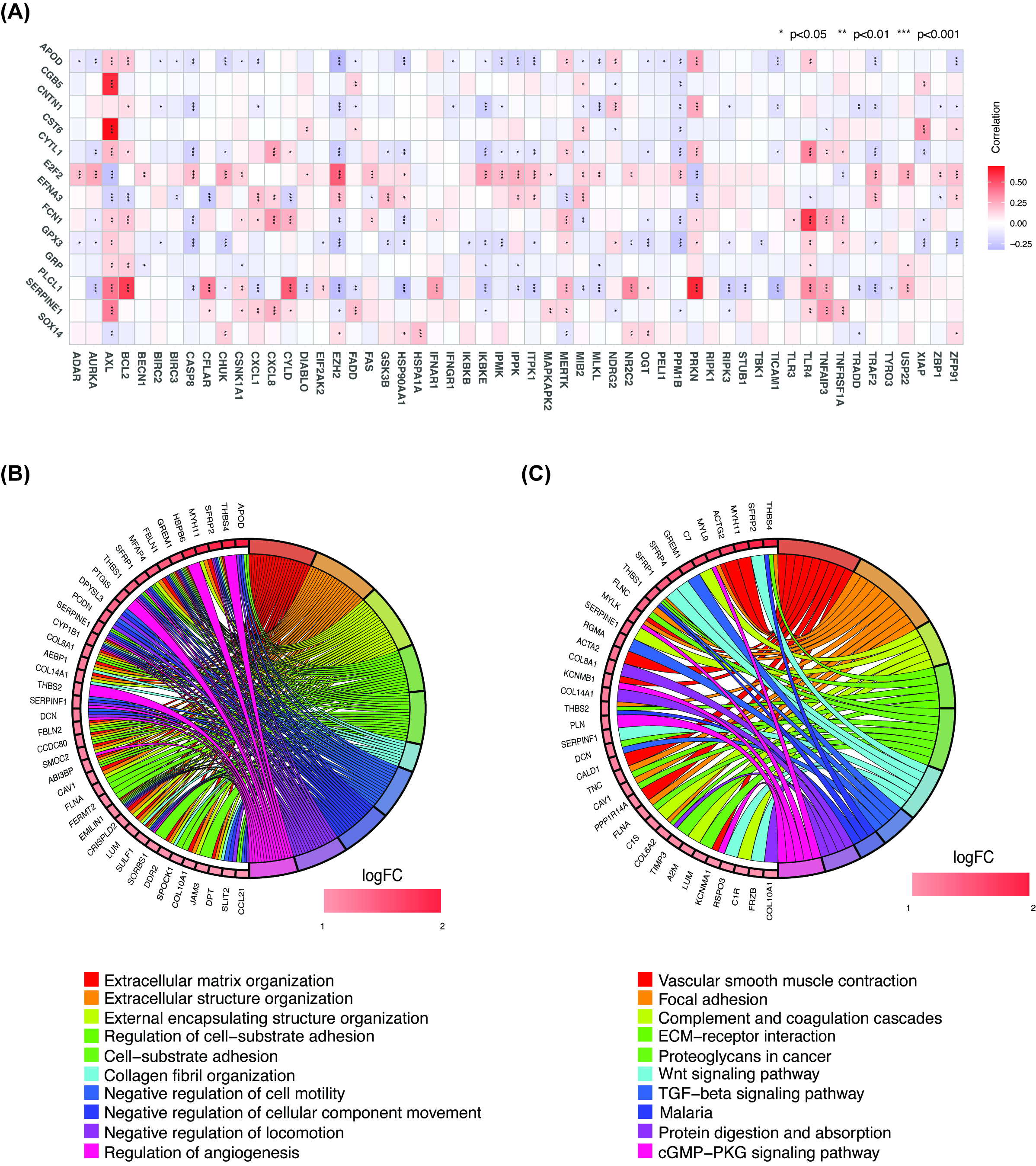

Supplement: Supplementary Figure 2 — Correlation and functional analysis of the risk groups. (A) Pearson’s correlation between individual NRGs (n = 55) and NRGPI (n = 13). P values are shown as: *P < 0.05; **P < 0.01; ***P < 0.001. (B) Circos plot depicting the enrichment of gene ontology (GO) terms (only biological process: BP) and (C) Kyoto Encyclopedia of Genes and Genomes (KEGG) pathways (increasing depth of the red indicate the more obvious differences; q-value: the adjusted p-value). [file Image_2.tif]

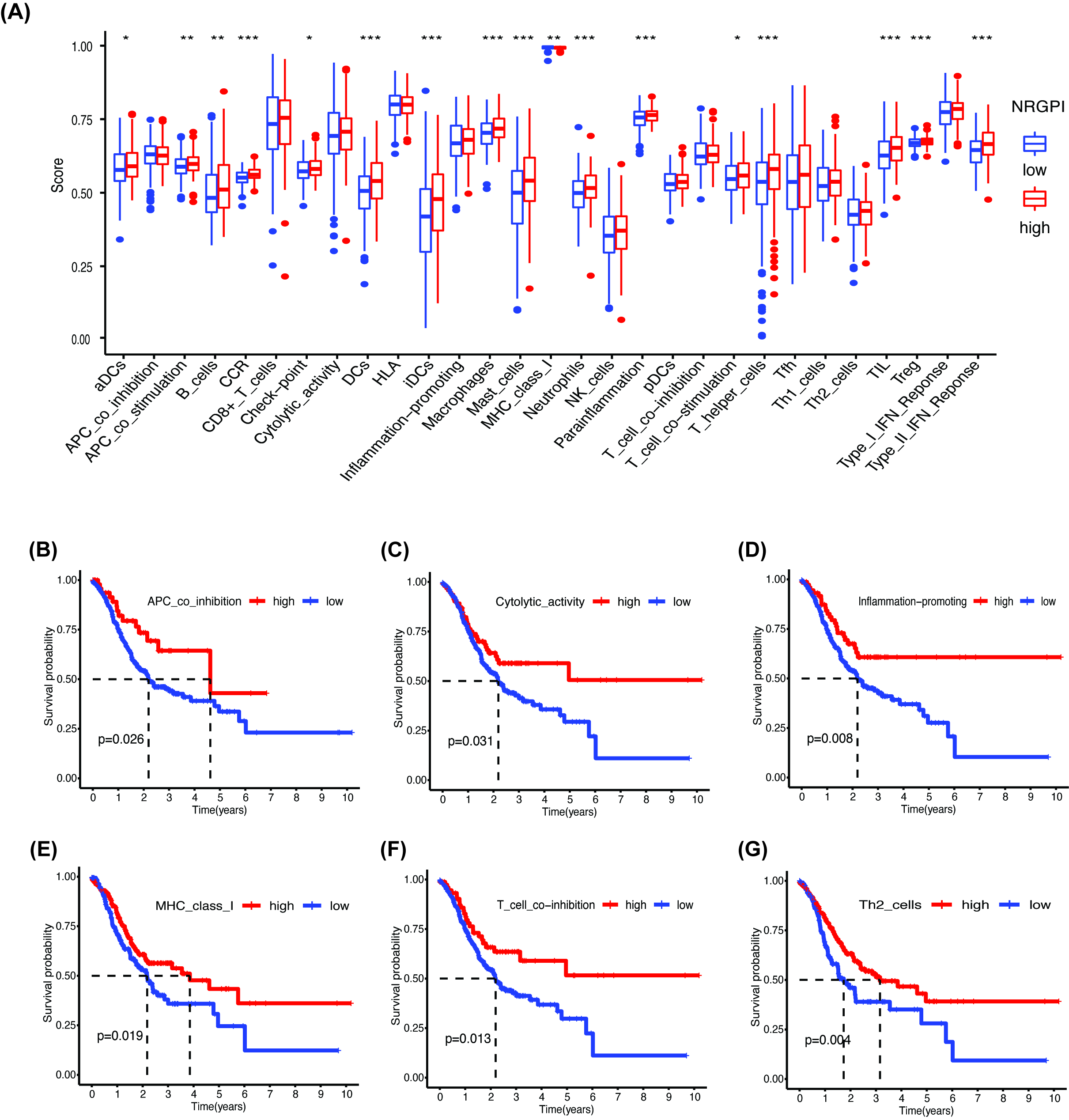

Supplement: Supplementary Figure 3 — Immune landscape of NRGPI subgroups based on the single sample gene set enrichment analysis (ssGSEA) scores. (A) Comparison of enrichment scores of 16 types of immune cells and 13 immune-related pathways in the GEO cohort between NRGPI-High and NRGPI-Low subgroups. (B–F) Kaplan-Meier curves for survival difference between TCGA patients with high and low-activation of immune-related pathways. (G) Kaplan-Meier curves for survival difference between TCGA patients with high and low- infiltration of Th2 cells. P values are shown as: *P < 0.05; **P < 0.01; ***P < 0.001. [file Image_3.tif]

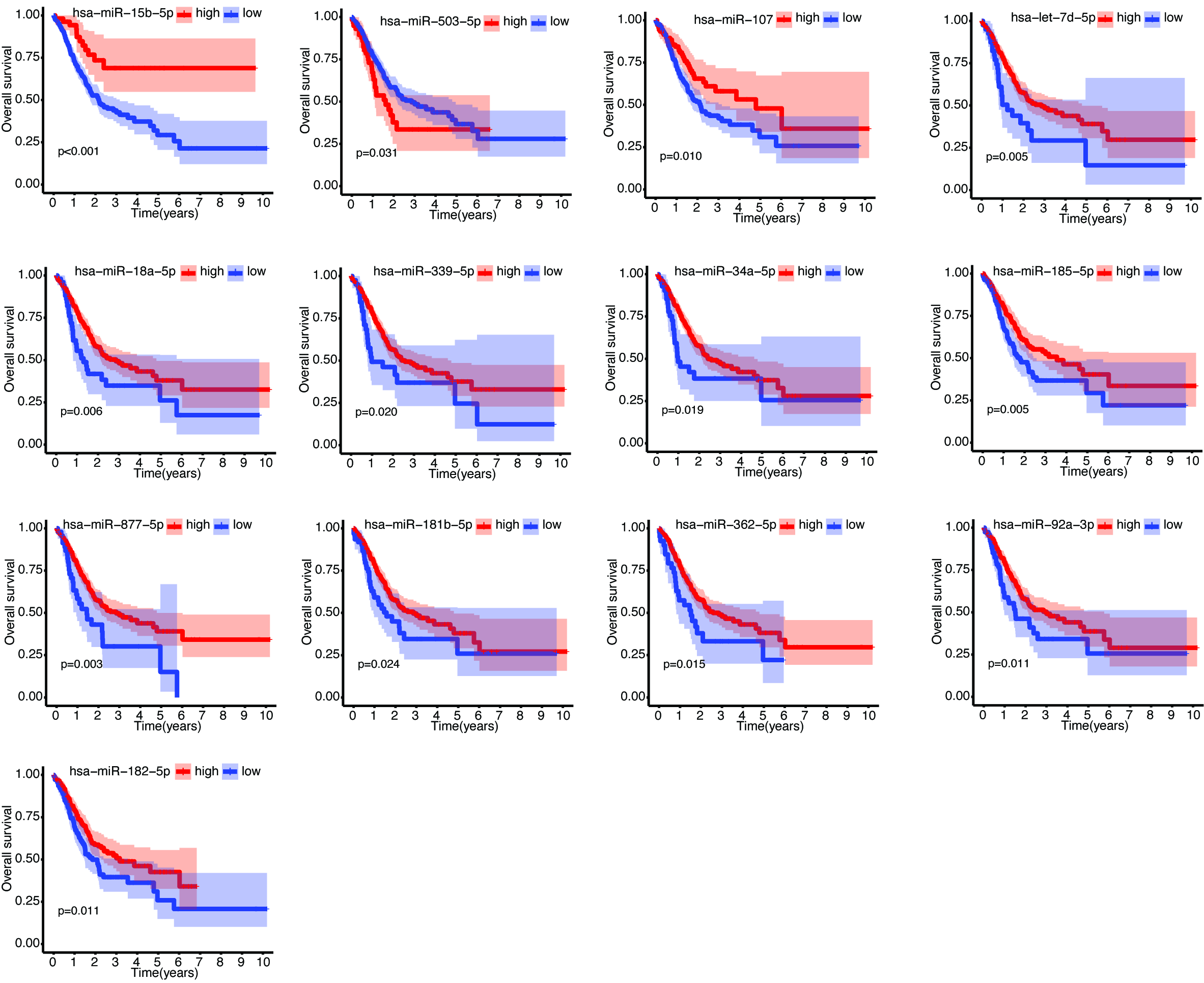

Supplement: Supplementary Figure 4 — Kaplan-Meier curves of survival analysis for miRNA targets of NRGPI oncogenes. [file Image_4.tif]

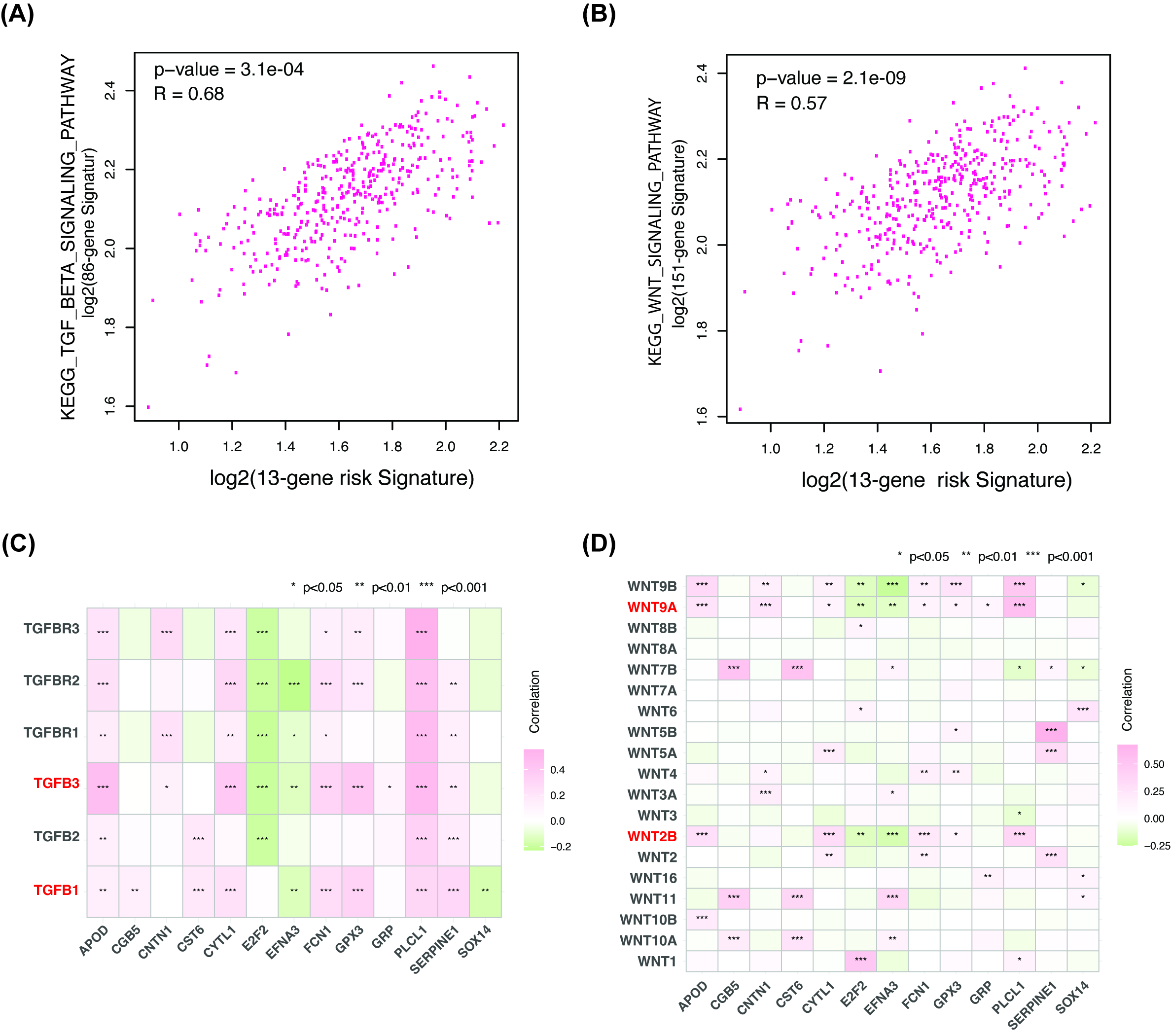

Supplement: Supplementary Figure 5 — Association of NRGPI and cancer-associated pathways (B) Spearman’s correlation between NRGPI (13 risk genes) signature and KEGG TGF-β signaling pathway (86-gene signature) in TCGA STAD cohort. (B) Spearman’s correlation between NRGPI (13 risk genes) signature and KEGG WNT signaling pathway (151-gene signature) in TCGA STAD cohort. (C) Pearson’s correlation between individual NRGPI (n=13) and markers of TGF-β signaling pathway in TCGA STAD cohort. P values are shown as: *P < 0.05; **P < 0.01; ***P < 0.001. (B) Pearson’s correlation between individual NRGPI (n=13) and markers of WNT signaling pathway in TCGA STAD cohort. P values are shown as: *P < 0.05; **P < 0.01; ***P < 0.001. [file Image_5.tif]

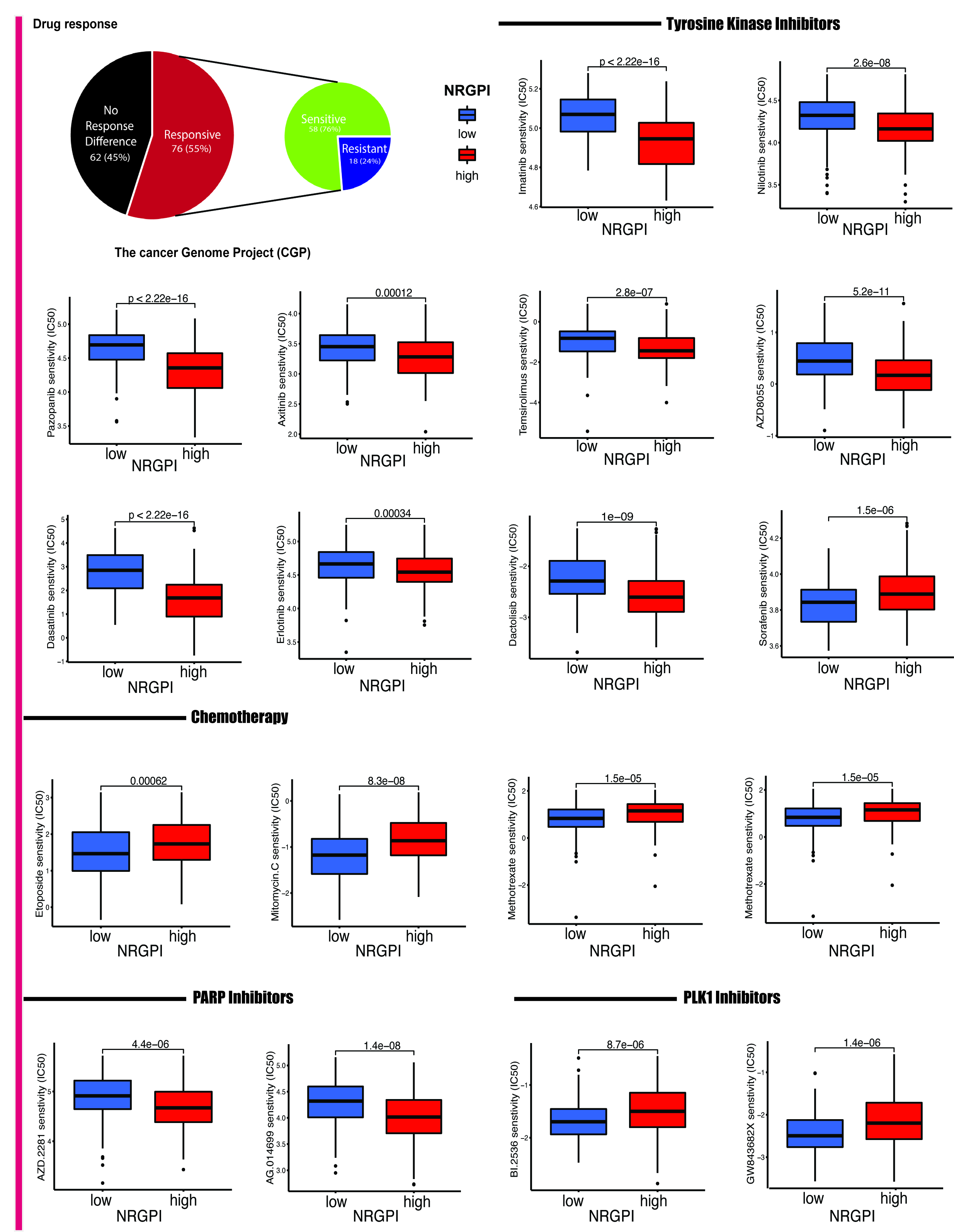

Supplement: Supplementary Figure 6 — Prediction of drug sensitivities of NRGPI-High and NRGPI-Low subgroups in TCGA cohort. [file Image_6.tif]
